# A Novel Stochastic Multi-Scale Model of *Francisella tularensis* Infection to Predict Risk of Infection in a Laboratory

**DOI:** 10.3389/fmicb.2018.01165

**Published:** 2018-07-06

**Authors:** Jonathan Carruthers, Martín López-García, Joseph J. Gillard, Thomas R. Laws, Grant Lythe, Carmen Molina-París

**Affiliations:** ^1^Department of Applied Mathematics, School of Mathematics, University of Leeds, Leeds, United Kingdom; ^2^Defence Science and Technology Laboratory, Salisbury, United Kingdom

**Keywords:** *Francisella tularensis*, Markov process, multi-scale model, dose response probability, mean response time, zonal ventilation model

## Abstract

We present a multi-scale model of the within-phagocyte, within-host and population-level infection dynamics of *Francisella tularensis*, which extends the mechanistic one proposed by Wood et al. (2014). Our multi-scale model incorporates key aspects of the interaction between host phagocytes and extracellular bacteria, accounts for inter-phagocyte variability in the number of bacteria released upon phagocyte rupture, and allows one to compute the probability of response, and mean time until response, of an infected individual as a function of the initial infection dose. A Bayesian approach is applied to parameterize both the within-phagocyte and within-host models using infection data. Finally, we show how dose response probabilities at the individual level can be used to estimate the airborne propagation of *Francisella tularensis* in indoor settings (such as a microbiology laboratory) at the population level, by means of a deterministic zonal ventilation model.

## 1. Introduction

*Francisella tularensis* is a gram-negative, facultative bacteria and the causative agent of tularemia (Oyston et al., 2004; Oyston, 2008). Due to its high infectivity and ability to cause a debilitating disease with the inhalation of as few as 10 organisms, *F. tularensis* has been classified as a category A bioterrorism agent by the Centers for Disease Control and Prevention (CDC). Following inhalation, bacteria are deposited in the lungs where, to begin with, they are primarily taken up by alveolar phagocytes through phagocytosis, as described by Hall et al. (2008). By escaping the *Francisella*-containing phagosome (FCP), bacteria enter into the cytosol of the phagocyte. *F. tularensis* can resist killing in the cytosol from reactive oxygen species (ROS) and can subsequently undergo multiple rounds of division within the host cell. Following this intracellular bacterial replication, the host phagocyte ruptures and dies, releasing its bacterial load back into the extracellular environment (Cowley and Elkins, 2011). For up to 72 h post-infection, *F. tularensis* is capable of preventing immune recognition. Therefore, it is important to understand how an individual may react to the infection, and when they develop tularemia.

Dose response models have been developed in an attempt to quantify the risk to a population associated with chemical and biological agents. However, unlike with chemical agents where the initial dose is the total amount available to cause a response, the ability of biological agents to reproduce post-infection means that the extent of replication within the host must be taken into account (Huang and Haas, 2009). Furthermore, since this timescale of infection is in the order of days, and since the window of opportunity for effective medical treatment is often limited, a better understanding of the infection timescale could provide valuable information to guide optimal treatment strategies. Attempts have therefore been made to incorporate time into such dose response models. Many of these original approaches involved adjusting existing dose response models, such as the classical exponential and beta-Poisson models, or probit analyses to allow for time dependency of the model parameters (Chen, 2007; Huang and Haas, 2009). However, by choosing convenient statistical distributions, the link between the dose response model and the underlying within-host biological mechanisms that govern the level of bacterial replication is tenuous. A stochastic mechanistic model is proposed by Pujol et al. (2009) for the within-host interaction dynamics between immune effector cells and pathogens, which takes into account both the total dose inhaled by the host and the total exposure period during which this dose is inhaled. It is also worth mentioning the work by Gillard et al. (2014), where a stochastic within-host computational model is proposed for the infection process, in the BALB/c mouse, following inhalational exposure to *Francisella tularensis SCHU S4*. By focusing on a compartmental agent based model, Gillard et al. (2014) consider the intracellular dynamics of a single infected phagocyte, and model the stages of bacterial replication and phagocyte rupture as a birth process with catastrophe, where the number of bacteria released in a single rupture event follows a geometric distribution. The average number of bacteria released is then estimated using the mean of this geometric distribution.

Another recent example is the Markov chain model described by Wood et al. (2014), which addresses these issues by considering the key interactions between *F. tularensis* bacteria and host (human) phagocytes within the lung space. Using the Markovian nature of the process, the probability and time for the total number of bacteria to reach some threshold can be computed, this threshold being identified as the necessary amount of bacteria needed for host illness onset. Despite this, fitting procedures are still used to obtain quantities, such as the time until a single infected phagocyte ruptures, which are required to parameterize the model. A particular limitation suggested by Wood et al. (2014) is the consideration of a deterministic (constant) time for the time to rupture of each infected phagocyte. This does not allow for modeling the experimentally observed variability in this time among different phagocytes, where in fact a log-normally distributed rupture time is predicted by Wood et al. (2014), but not explicitly incorporated into the model. Also, by using a deterministic approach to modeling the intracellular growth of *F. tularensis* bacteria, Wood et al. (2014) assume a constant number of bacteria released on rupture of any infected phagocyte, not accounting for the existing variability in the number of bacteria released by different phagocytes.

In this paper, an extension to the model described by Wood et al. (2014) is proposed. By incorporating a second, within-phagocyte, model into the existing within-host model, the stochastic intracellular dynamics of *F. tularensis* can be replicated. This can account for the log-normally distributed rupture time, leading to a rupture size probability distribution (i.e., number of bacteria released upon phagocyte rupture) which enables us to account for inter-phagocyte variability at the within-host level. Thus, the within-phagocyte model can be linked with the within-host model for the interaction between extracellular bacteria and susceptible phagocytes by means of the distribution of the number of bacteria released by a single infected phagocyte, obtained from analyzing the within-phagocyte model, which allows for varying phagocyte rupture sizes in the within-host model. In summary, this multi-scale model allows us to relax the assumption made by Wood et al. (2014) that a fixed number of bacteria is released from every single infected phagocyte on rupture. For both the within-host and within-phagocyte models, analytical approaches to calculate the summary statistics (dose response probability and mean time until response) defined by Wood et al. (2014) are outlined. However, by exploiting the structure of the resulting Markov processes, more efficient approaches than the methods proposed by Wood et al. (2014) are described here. Finally, a zonal ventilation model for the indoor airborne spread of *F. tularensis* is presented in order to illustrate how dose response probabilities at the individual level, computed from the within-host model, can be used in order to make predictions at the population level.

## 2. Materials and methods

In this section, our aim is to develop a multi-scale model for the infection dynamics of *F. tularensis* bacterium, by linking a within-phagocyte, a within-host and a population-level model. In section 2.1 we develop a stochastic within-phagocyte model for the infection dynamics of a single phagocyte by *F. tularensis*. We show how the log-normally distributed rupture time estimated by Wood et al. (2014) from experimental data (Lindemann et al., 2011), can be incorporated into this model, while maintaining the Markovian nature of the underlying stochastic process, and how first-step arguments allow one to compute the probability distribution of the total number of bacteria released by an infected phagocyte upon rupture. This distribution is used in section 2.2 to link the within-phagocyte model to the within-host model for the interaction between extracellular bacteria and phagocytes within the host. This within-host model accounts for inter-phagocyte variability in the amount of bacteria released upon rupture. The aim of the within-host model is to compute the probability of host response (in terms of the onset of symptoms), as well as the time to this response. Finally, we illustrate in section 2.3 how these dose response probabilities at the individual level might be used for predicting, at the population level, the number of individuals showing symptoms upon indoor release and airborne spread of *F. tularensis*, by means of a zonal ventilation model and under different ventilation settings in an hypothetical microbiology laboratory.

### 2.1. Within-phagocyte model

**Figure 1:**
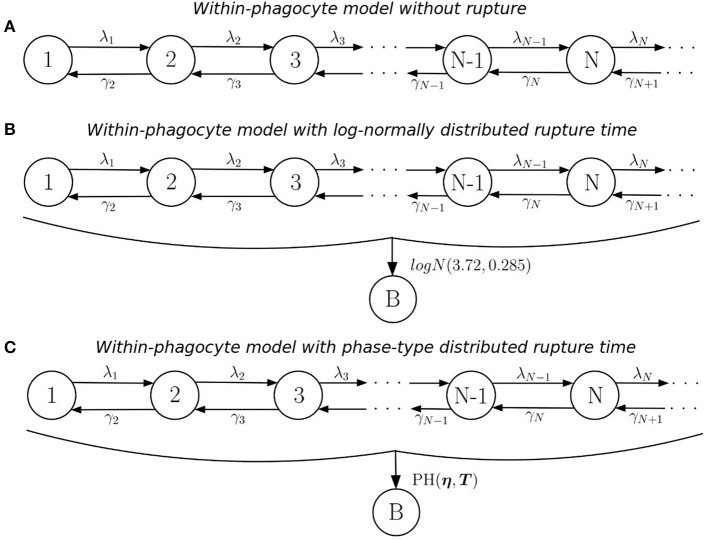
Within-phagocyte model. **(A)** Logistic growth process for the within-phagocyte replication of bacteria; **(B)** logistic growth process with log-normally distributed phagocyte rupture, moving the process to absorbing state *B*; **(C)** approximation of the process in **(B)** by using a *PH*(**η**, ***T***) distribution for the rupture time.

The first level of the multi-scale model is a within-phagocyte model, used to replicate the dynamics of an *F. tularensis* bacterium after it has been *ingested* by a host phagocyte, assuming that the bacterium escapes the FCP, entering into the cytosol and starting replication. Phagocytosis leading to successful bacterial killing will be considered in the within-host model, and is not analyzed here. This includes the replication of bacteria within the cytosol, and the subsequent rupturing and death of the phagocyte (Cowley and Elkins, 2011). These stages of the intracellular life-cycle can be modeled using a continuous-time stochastic process X={X(t): t≥0} that follows the structure of a birth-and-death process with catastrophe (Karlin and Tavaré, 1982; Di Crescenzo et al., 2008), where *X*(*t*) is the number of bacteria within the phagocyte at time *t* ≥ 0. In particular, replication and death of bacteria within the phagocyte can be modeled as a stochastic logistic growth process over states in ℕ = {1, 2, … }, representing the number of bacteria contained within the cytosol (see Figure 1A). Birth and death rates for state *n* ∈ ℕ are obtained by following arguments by Allen (2003, section 6.8), where we assume that each bacterium replicates independently of all others at rate λ, so that:

(1) $$\begin{array}{l} \lambda_{n}=\{\begin{array}{ll} \frac{\lambda (C-1)}{C} & \text{if }n=1,\\ \lambda n & \text{otherwise,} \end{array}\;\gamma_{n}=\{\begin{array}{ll} 0 & \text{if }n=1,\\ \frac{\lambda n^{2}}{C} & \text{otherwise}. \end{array} \end{array}$$

We denote by λ [*hours*^−1^] the per bacterium birth rate, and by *C* [*bacteria*] the carrying capacity of the population of intracellular bacteria within a single phagocyte, which represents limitation of nutrients necessary for replication, such as iron or tryptophan (Jones et al., 2012). The decision to assume logistic growth for the intracellular bacteria reflects the competition for *resources* within the phagocyte. The rate γ_1_ is set to zero since only phagocytes experiencing an effective long-term bacterial infection (and within-phagocyte replication) are later considered in the within-host model. The initial state of the process X corresponds to the number of bacteria taken up by a phagocyte. Experimental evidence by Golovliov et al. (2003) suggests that the uptake of *F. tularensis* is relatively ineffective in monocytic cells so that, during the initial phase of the infection, on average only one or two intracellular bacteria per cell were observed. Thus, we assume that a single phagocyte will take up a single bacterium, hence the process X will always begin in state *X*(0) = 1. Once infected, the possibility of the phagocyte taking up more bacteria is neglected (Wood et al., 2014), and an increase in its bacterial load is solely due to the replication of the bacterium initially ingested. We refer the reader to the Supplementary Material where the impact of this assumption is further explored.

The number of bacteria released upon rupture of an infected phagocyte will depend on the stochastic dynamics of this logistic growth process, as well as on the actual time when this rupture takes place. In order to describe this rupture event, we consider additional transitions to an absorbing (rupture) state, *B*, from any of the transient states in ℕ, as shown in Figure 1B. The rate at which this rupture event occurs is assumed to be independent of the number of bacteria within the phagocyte. This is based on the fact that bacterial escape into the cytosol has been shown to be both essential and sufficient for triggering caspase-3 activation, which is the mechanism thought to induce cell death (Santic et al., 2010). In fact, a recent experimental study by Brock and Parmely (2017) shows that cell death does not require high bacterial burden, nor does a large number of intracellular bacteria ensure that phagocyte rupture would result soon. This implies that X can be thought of as a stochastic birth-and-death process where *t* = 0 marks the start of a “clock” that counts up toward the time of rupture of the phagocyte. At this moment, X immediately transitions into state *B*, from whichever of the transient states this may be, this state accounting for the number of bacteria released upon rupture (i.e., the rupture size). By fitting a deterministic model to experimental data, Wood et al. (2014) found that the time *T*^*rupture*^ taken for an infected phagocyte to rupture is log-normally distributed, *T*^*rupture*^ ~ *logN*(3.72, 0.385), so that the average rupture time is *E*[*T*^*rupture*^] = 44.4 h. Instead of incorporating this log-normally distributed time in the within-phagocyte model, Wood et al. (2014) consider a deterministic logistic growth process for the amount of bacteria within the phagocyte. Finally, Wood et al. (2014) set the number of bacteria released to be equal to the amount of bacteria in this logistic growth process at time *Median*[*T*^*rupture*^] hours (i.e., by considering *Median*[*T*^*rupture*^] and neglecting the actual distribution of the random variable *T*^*rupture*^), which leads to a constant and deterministic number of bacteria released for any infected phagocyte.

**Figure 2:**
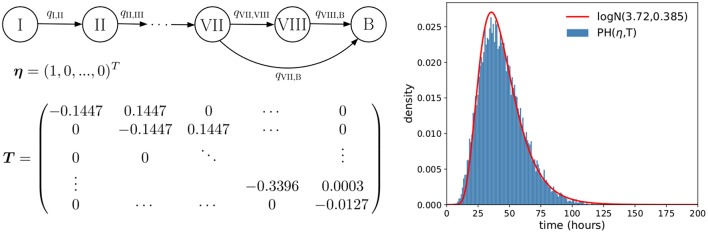
Phase-type approximation of the log-normally distributed rupture time. **(Left)** A depiction of the one-dimensional Markov process W (see Supplementary Material) associated with the *PH*(**η**, ***T***) distribution considered, so that the time to reach state *B* approximately follows $T^{rupture}\overset{approx.}{\sim }logN\left(3.72,0.385\right)$. **(Right)** Plot showing how accurately the *PH*(**η**, ***T***) distribution approximates the desired log-normal distribution.

If a log-normal distribution of *T*^*rupture*^ is used in our model to compute the probability distribution of the number of bacteria released upon phagocyte rupture, this leads to the process described in Figure 1B. However, by considering a log-normally distributed inter-event time in the stochastic process, the resulting process X in Figure 1B is no longer Markovian. In order to address this difficulty, we propose to approximate this log-normally distributed rupture time *T*^*rupture*^ ~ *logN*(3.72, 0.385) by a phase-type (PH) distribution, $T^{rupture}\overset{approx.}{\sim }PH\left(\eta ,\text{T}\right)$, since the family of phase-type distributions is dense within the family of continuous non-negative distributions (He, 2014). This leads to the process shown in Figure 1C. In the Supplementary Material, we explain in detail how one can approximate this log-normal distribution by an approximate phase-type distribution, which depends on parameters **η** (a vector) and **T** (a matrix). The resulting estimated parameters **η** and **T** obtained for a PH distribution which approximates the *logN*(3.72, 0.385) distribution, as well as a graphical representation of this approximation, are reported in Figure 2.

Once the log-normal distribution for the rupture time has been approximated by a PH distribution, the resulting within-phagocyte stochastic process X in Figure 1C is Markovian, and the probability distribution of the number of bacteria *R* released upon rupture can be analytically computed (see Supplementary Material). The probability distribution of *R*, defined in terms of the following probabilities

(2) $$\begin{array}{l} R_{k}=\mathbb{P}\left(R=k\right)\;=\text{probability that the infected phagocyte}\\ \text{ releases }k\text{ bacteria upon rupture}, \end{array}$$

is used in section 2.2 to incorporate inter-phagocyte variability (in the amount of bacteria released upon phagocyte rupture) in the within-host infection dynamics.

### 2.2. Within-host model

The within-host model proposed here is a birth-death-rupture model that replicates the dynamics of *F. tularensis* within the lung, following inhalation of some initial quantity of bacteria (initial dose), and is largely based on the original model by Wood et al. (2014). Within the lung, bacteria can be killed by host immune cells or ingested by host phagocytes. In the latter case, the phagocyte might kill the corresponding bacterium (e.g., if the phagocyte is activated), or this bacterium can escape the FCP and enter into the cytosol, resulting in rapid proliferation of the bacteria and the subsequent rupture and death of the phagocyte, as described by the within-phagocyte model. Three events are therefore included in the within-host model, as well as their effect on the total population of bacteria and the number of infected phagocytes, and are detailed as follows:

- **Phagocytosis and bacterial survival (rate** α > 0[*hours*^−1^]**)**: this phagocytosis event refers to the phagocytosis and intracellular survival of a bacterium; that is, to phagocytosis resulting in bacterial escape from the FCP, and in the subsequent events represented by the within-phagocyte model.
- **Extracellular bacterial death (rate** μ > 0[*hours*^−1^]**)**: host defense mechanisms such as the complement system, antibodies, natural killer cells, activated phagocytes and antimicrobial peptides directly contribute to the killing of extracellular bacteria (Jones et al., 2012). These methods of killing, including phagocytosis with successful intracellular bacterial killing, are collectively represented in the within-host model as this single event, with rate μ.
- **Rupture of infected phagocytes (rate** δ = *Median*[*T*^*rupture*^]^−1^[*hours*^−1^]**)**: following phagocytosis of bacteria that results in their survival and intracellular proliferation, infected phagocytes rupture and die. The distribution of the number of bacteria released, computed by means of the within-phagocyte model, is incorporated here in terms of probabilities *R*_*k*_. This then accounts for an addition to the original model by Wood et al. (2014), allowing for inter-phagocyte variability in the rupture size.

In this way, the within-phagocyte model in section 2.1 allows one to represent the intracellular bacterial dynamics for bacteria surviving the phagocytosis event, escaping the FCP and entering into the cytosol, eventually leading to phagocyte rupture and bacterial release. Phagocytosis leading to successful bacterial killing is one of the several mechanisms described above leading to bacterial death at the within-host level. Furthermore, intracellular bacterial replication is not explicitly considered in the within-host model, since one bacterium is considered per infected phagocyte. Once rupture of an infected phagocyte occurs, the number of bacteria released to the extracellular environment is given by the rupture size distribution computed from the within-phagocyte model. Given that *R*_*k*_ is the probability that an infected phagocyte, initially infected by a single bacterium, releases *k* bacteria on rupture, the rate at which an infected phagocyte ruptures releasing *k* bacteria in the within-host model is then given by δ*R*_*k*_. We note that since $\overset{\infty }{\underset{k=1}{\sum }}R_{k}=1$, δ can be interpreted as the total rate of rupture of a single phagocyte.

**Figure 3:**
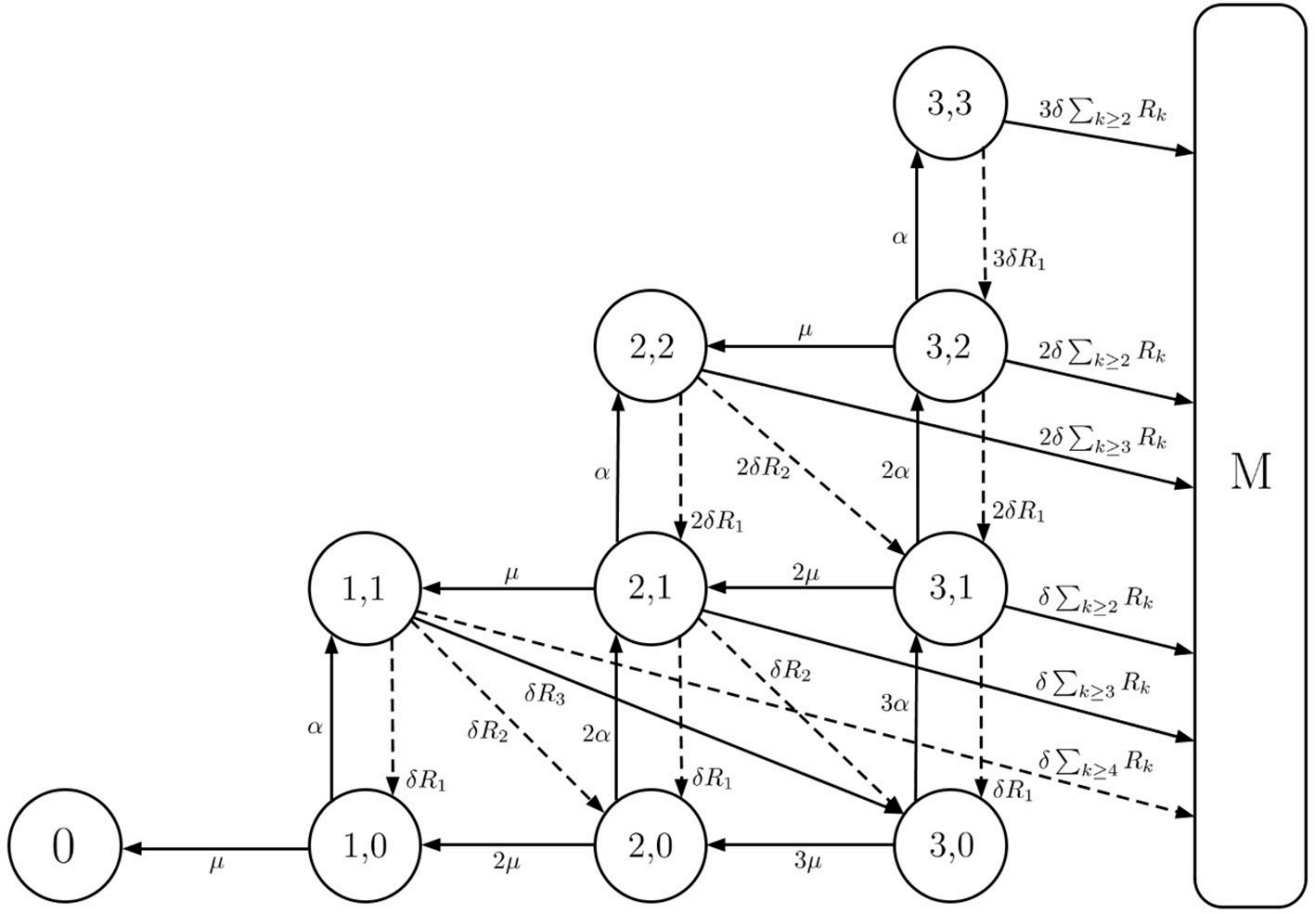
Within-host model with inter-phagocyte variability. A depiction of the extended two-dimensional Markov chain with *M* = 4. State (*i, j*) represents *i* extracellular bacteria and bacteria-containing phagocytes, and *j* bacteria containing phagocytes. The rates of rupture, phagocytosis, and death of extracellular bacteria are δ > 0, α > 0 and μ > 0, respectively. In our model, each rupturing phagocyte releases *k* bacteria with probability *R*_*k*_. Solid arrows represent the events allowed in the original model (Wood et al., 2014), where each rupturing phagocyte always releases *G* = 3 (for illustrative purposes; *G* = 358 in the original model by Wood et al., 2014) bacteria upon rupture. Dashed arrows are an addition in our model, and account for inter-phagocyte variability in the rupture size.

The within-host model can be described using a continuous-time two-dimensional Markov process Y={Y(t)=(B(t),P(t)):t≥0}, where *B*(*t*) denotes the total number of extracellular bacteria and bacteria-containing phagocytes at time *t* ≥ 0, and *P*(*t*) represents the number of infected phagocytes at time *t* ≥ 0, *B*(*t*) ≥ *P*(*t*) for any time instant *t* ≥ 0. An initial state of Y given by **Y**(0) = (*k*, 0) represents that *k* is the number of bacteria initially inhaled by the individual (initial dose), and there are 0 infected phagocytes. When the total population of bacteria reaches a threshold *M* ∈ ℕ, a response is assumed to occur and reflects the onset of symptoms in the infected individual (Wood et al., 2014). This state, *M*, referred to as the response state, is one of two absorbing states of Y; the other is state 0 and represents the clearance of infection without reaching this response threshold. A depiction of the model is provided in Figure 3.

Two summary statistics of interest in the within-host model are the probability of response and the mean response time. For each of these, an efficient analytic approach for their exact computation can be found in the Supplementary Material. In particular, we define π_(*i,j*)_ as the probability of response given the initial state **Y**(0) = (*i, j*)

(3) $$\begin{array}{l} \pi_{\left(i,j\right)}=\underset{t\to +\infty }{\lim}\mathbb{P}\left(\text{Y}\left(t\right)=M|\text{Y}\left(0\right)=\left(i,j\right)\right),\;0\leq j\leq i\leq M-1. \end{array}$$

By applying first-step arguments, the following recursive formula for π_(*i,j*)_ may be obtained

(4) $$\begin{array}{l} \pi_{\left(i,j\right)}=\frac{1}{(i-j)(\alpha +\mu )+\delta j}[(i-j)\left(\alpha \pi_{\left(i,j+1\right)}+\mu \pi_{\left(i-1,j\right)}\right)\\ +\;\delta j\left(\sum_{k=1}^{M-i}R_{k}\pi_{\left(i+k-1,j-1\right)}+\sum_{k\geq M-i+1}R_{k}\right)], \end{array}$$

for 0 ≤ *j* ≤ *i* ≤ *M*−1, with the boundary condition π_(0, 0)_ = 0 representing that the probability of response is equal to zero if the recovery state is reached. A detailed derivation of this expression, as well as an algorithmic solution to the previous equations, are provided in the Supplementary Material.

One may define the mean time until the infected host responds in terms of the onset of symptoms. This can be done by choosing a threshold in the total number of extracellular bacteria equal to *M*, and considering the time to get to *M*, *T*_(*i,j*)_ = *inf*{*t* ≥ 0:*B*(*t*) = *M* | **Y**(0) = (*i, j*)}. Since absorption into the response state *M* is not certain, there is no guarantee that the time to response, *T*_(*i,j*)_, will be finite (i.e., *T*_(*i,j*)_ = +∞ if the individual recovers without reaching the threshold state *M*, while *T*_(*i,j*)_ < +∞ if this threshold is reached, leading to the onset of symptoms). Thus, one may compute the *restricted* mean response time, after which the *conditioned* mean response time can be obtained. That is, if *T*_(*i,j*)_ denotes the time to reach state *M* provided that **Y**(0) = (*i, j*), then the restricted and conditioned mean response times are given respectively by

(5) $$\begin{array}{l} r_{\left(i,j\right)}=\text{E}\left[T_{\left(i,j\right)}1_{T_{\left(i,j\right)}<+\infty }\right],\\ m_{\left(i,j\right)}=\text{E}\left[T_{\left(i,j\right)}|T_{\left(i,j\right)}<+\infty \right]=\frac{r_{\left(i,j\right)}}{\pi_{\left(i,j\right)}}\;0\leq j\leq i\leq M-1, \end{array}$$

where 1_*A*_ is equal to 1 if *A* is satisfied and 0 otherwise. Following a first-step analysis, a recursive formula for the scalar quantities *r*_(*i,j*)_ is given by

(6) $$\begin{array}{l} r_{\left(i,j\right)}=\frac{1}{(\alpha +\mu )(i-j)+\delta j}[(i-j)(\alpha r_{\left(i,j+1\right)}+\mu r_{\left(i-1,j\right)})+\delta j\sum_{k=1}^{M-i}R_{k}r_{\left(i+k-1,j-1\right)}\\ +\frac{(i-j)(\alpha \pi_{\left(i,j+1\right)}+\mu \pi_{\left(i-1,j\right)})+\delta j\left(\sum_{k=1}^{M-i}R_{k}\pi_{\left(i+k-1,j-1\right)}+\sum_{k\geq M-i+1}R_{k}\right)}{(i-j)(\alpha +\mu )+\delta j}],\; \end{array}$$

for 0 ≤ *j* ≤ *i* ≤ *M*−1, with the boundary condition *r*_(0, 0)_ = 0 representing the restricted time to a response if the recovery state is reached. Similar arguments to those used for computing the dose response probabilities, and described in the Supplementary Material, may be used for solving Equation (6) in an algorithmic and matrix-oriented way.

### 2.3. Population-level model

With a multi-scale model of *F. tularensis* infection that captures both the intracellular and within-host dynamics, we can now formulate a population scale model. At the population level, outbreaks of tularemia, as a result of infection by *F. tularensis*, have been declared in microbiology laboratories (Shapiro and Schwartz, 2002). This is directly related to the fact that diagnosis of tularemia requires a high level of suspicion, since the disease often presents with non-specific symptoms and non-specific results of routine laboratory tests (Report, 2008). Because *F. tularensis* is a risk to laboratory personnel, clinicians are required to notify the laboratory when tularemia is suspected in a given patient, and if this is not notified, an outbreak can occur within the laboratory when manipulating the contaminated samples, as in the outbreak reported by Shapiro and Schwartz (2002). In particular, this notification allows for manipulation of the corresponding samples to be carried out under strict control measures, such as Biosafety Level 2 (BSL-2) or BSL-3 conditions (Report, 2008). If proper control measures are not taken when manipulating these samples, or if an accident occurs, *F. tularensis* can be released to the air, triggering its airborne dispersal and spread. Specific high-risk sample manipulation activities that have been identified in the literature are centrifuging, grinding or vigorous shaking (Report, 2008).

**Figure 4:**
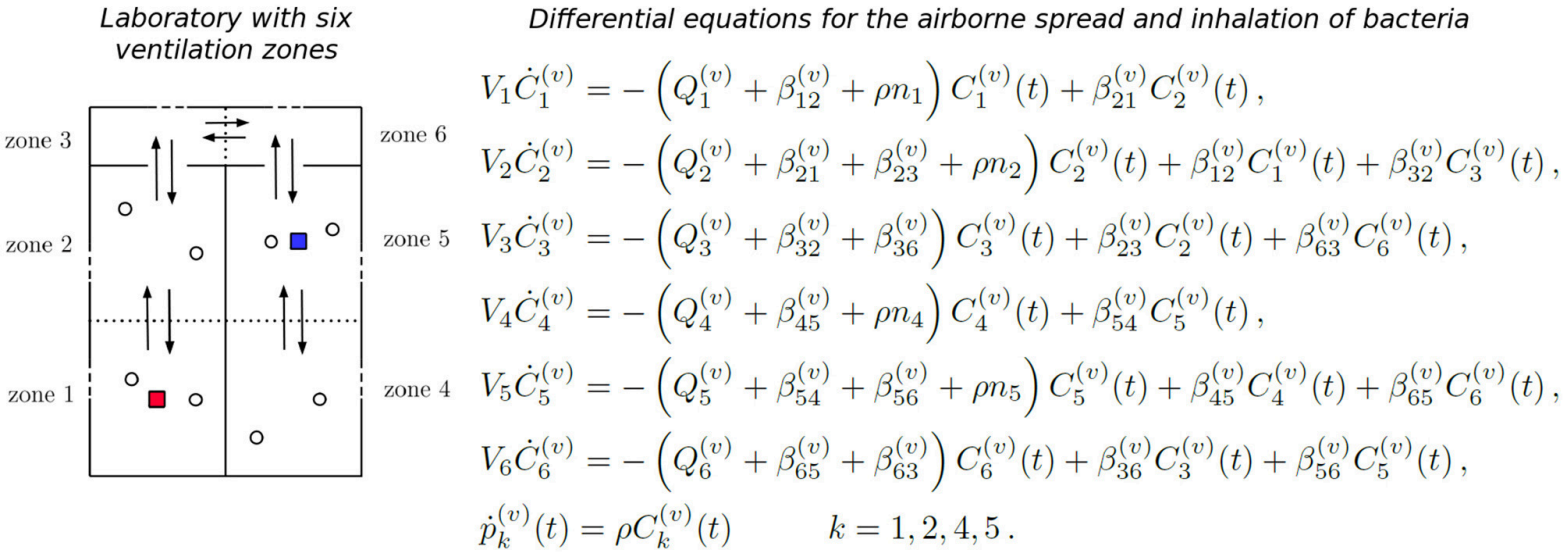
Zonal ventilation model for the airborne spread of bacteria within a microbiology laboratory. **(Left)** A diagram showing the setup of two rooms and a corridor within a laboratory, split into six ventilation zones. Dotted lines represent the partitions of rooms, arrows between zones show potential airflow, which depends on the particular ventilation setting *v* considered, and dashed lines represent potential extract ventilation systems within each zone. Individuals are represented by circles, and the red and blue squares indicate two potential locations of the initial bacterial release. **(Right)** The system of ODEs that governs the airborne spread of bacteria across the ventilation zones, and the inhalation of these bacteria by individuals, for a particular ventilation setting *v*. Concentration of bacteria at zone *j*, *C*_*j*_(*t*), increases with flow of air from neighboring zones (rates $\beta_{ij}^{\left(v\right)}$) and decreases due to inhalation (rate ρ), flow of air to neighboring zones (rates $\beta_{ji}^{\left(v\right)}$) and extraction (rate $Q_{j}^{\left(v\right)}$). The cumulative amount of bacteria inhaled by each individual at zone *j* is denoted by $p_{j}^{\left(v\right)}\left(t\right)$.

Recent work has been carried out for the indoor airborne spread of pathogens while taking into account the ventilation regime in place at the facility under analysis, such as the airborne spread of bacteria in health care facilities (Liao et al., 2005). For these scenarios, zonal ventilation models that are able to link the airflow dynamics within the facility and the epidemic dynamics at the population level are extremely useful (Noakes and Sleigh, 2009; López-García et al., under review). We consider here the scenario of a laboratory consisting of two rooms joined by a corridor, and where a given *ventilation setting*, in terms of the airflow dynamics, is in place (see Figure 4). We consider that a fixed amount of bacteria is released at time *t* = 0 in a given room. This could represent some high-risk manipulation of a contaminated sample or any accident causing airborne release of *F. tularensis* bacteria, which we assume here passes unnoticed for the staff (Shapiro and Schwartz, 2002). Our aim is to estimate, for any given ventilation setting and any possible spatial position of the release location within the laboratory, the total number of individuals who would develop symptoms in the near future.

We propose here to follow the approach introduced by Noakes and Sleigh (2009), recently extended by López-García et al., (under review), where a system of ordinary differential equations (ODEs) is used to model the concentration of *F. tularensis* in the air in the different spatial compartments of the laboratory. In particular, a ventilation regime is defined by splitting this laboratory into *ventilation zones*, where the main assumption is that the air is well-mixed in each zone, but that airflow imbalances across the different zones can lead to different pathogen concentrations in the air at each zone (Noakes and Sleigh, 2009). Therefore, individuals in the same ventilation zone are assumed to have equal probability of inhaling the *F. tularensis* bacteria. Airflow dynamics could be further refined by considering a larger amount of ventilation zones. If *C*_*i*_(*t*) [*bacteria*·*m*^−3^] denotes the concentration of bacteria in the air in zone *i* at time *t*, and *p*_*i*_(*t*) [*bacteria*] is the total amount of bacteria inhaled by each individual in this zone up to time *t*, then *C*_*i*_(*t*) and *p*_*i*_(*t*) satisfy the system of ODEs given in Figure 4. Here, *V*_*i*_ [*m*^3^] denotes the volume of zone *i*, *Q*_*i*_ [*m*^3^·*min*^−1^] is the rate at which air is extracted from zone *i* by the ventilation system, β_*ij*_ [*m*^3^·*min*^−1^] is the rate at which air flows from zone *i* to zone *j*, *n*_*i*_ is the number of individuals in zone *i*, and ρ [*m*^3^·*min*^−1^] is the pulmonary rate, or the rate at which individuals inhale air (Noakes and Sleigh, 2009). We set *n*_*i*_ = 2 for *i* ∈ {1, 2, 4, 5} to represent two individuals working in each of these zones during the bacterial release, where the propagation occurs in the timescale of minutes (see section 3), and *n*_*i*_ = 0 for *i* ∈ {3, 6} (i.e., corridor areas).

We propose to link the dose response probabilities obtained from the within-host model with this zonal ventilation model, by considering that the steady state value of *p*_*i*_(*t*) is equal to the total dose that an individual in zone *i* inhales. Thus, we implicitly assume that the timescale at which *p*_*i*_(*t*) reaches equilibrium (minutes, see section 3), is significantly shorter than the timescale of the within-host infection dynamics (days, see section 3), so that *lim*_*t* → +∞_*p*_*i*_(*t*) can be considered as the initial dose for individuals in zone *i*. We note that the differential equations in Figure 4 depend on the rates of the ventilation setting under analysis, and on the initial conditions *C*_*i*_(0), 1 ≤ *i* ≤ 6 (related to where the bacterial release occurs in the first place). In section 3, we consider different ventilation settings and potential initial locations of the bacterial release.

## 3. Parameter values

In this section, we discuss how to calibrate our within-phagocyte and within-host models from data. We also consider different ventilation settings for the population model, according to values reported by Noakes and Sleigh (2009) for the airborne spread of bacteria within a health care facility.

### 3.1. Within-phagocyte model

In order to use the within-phagocyte model described in section 2 to compute the rupture size distribution of any given infected phagocyte, parameters λ and *C* must first be estimated for the logistic growth process in Figure 1 for the within-phagocyte bacterial replication. We do so, making use of experimental data of the number of intracellular bacteria within a phagocyte (Lindemann et al., 2011). In this experiment, measurements of the number of intracellular bacteria were only considered for phagocytes that were still alive and had not ruptured (Lindemann et al., 2011). Therefore, we obtain estimations for λ and *C* by calibrating the logistic growth process in Figure 1A, where rupture events are neglected.

A sequential Approximate Bayesian Computation (ABC) method is used to get estimations for these parameters. When implementing the ABC method, unknown parameter values are sampled from a *prior* distribution, and model predictions (e.g., number of intracellular bacteria at different time instants) are obtained for these parameter values. Once these predictions are in hand, one can compare these model predictions with experimental data by using a particular distance measure, and accept or reject these sampled parameter values depending on this distance being below or above a given threshold ϵ. Accepted sampled parameter values lead to a *posterior* distribution for the corresponding parameters (Kypraios et al., 2017).

**Figure 5:**
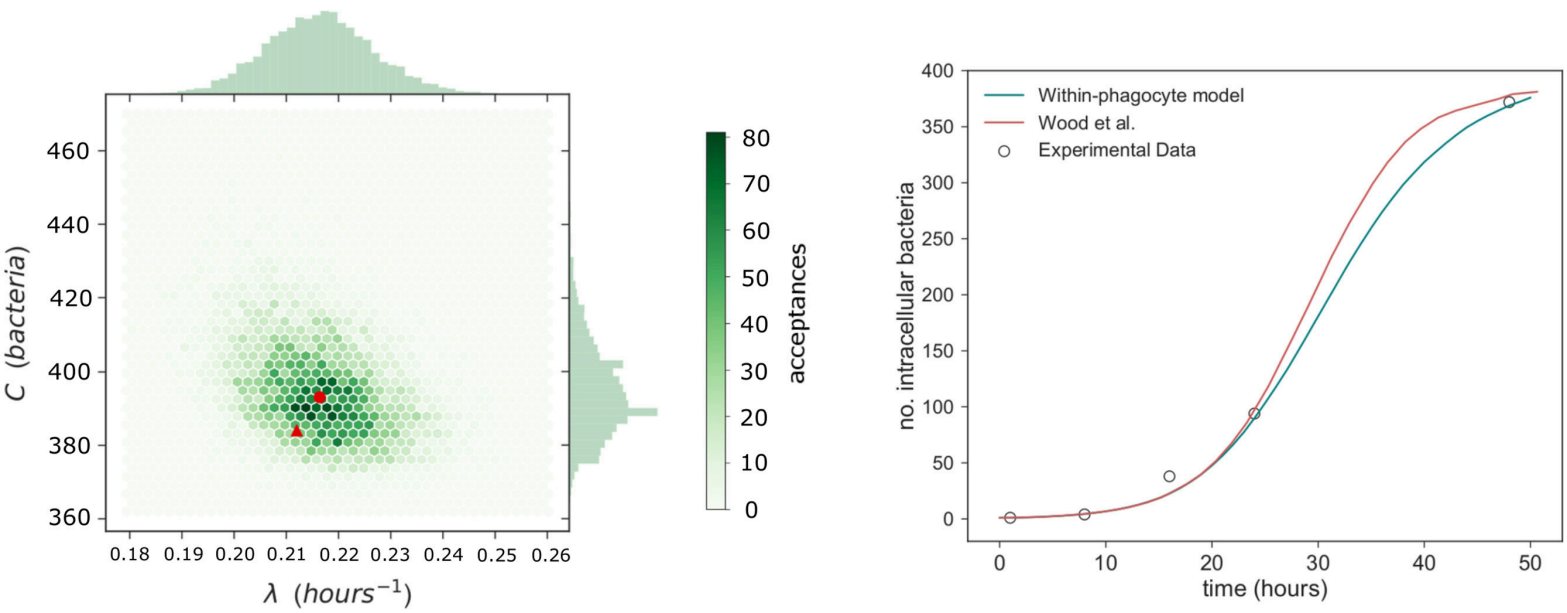
Within-phagocyte parameter estimation. **(Left)** A bivariate histogram of λ and *C* accepted values as a result of the ABC method, with median values marked with a red circle. Values (λ, *C*) estimated by Wood et al. (2014) are reported with a red triangle. **(Right)** Number of bacteria within an infected phagocyte through time, predicted from our model (blue curve) using median posterior values for (λ, *C*), and compared to the theoretical predictions by Wood et al. (2014) (red curve), and experimental data by Lindemann et al. (2011) (circles).

We consider as prior distributions for each parameter λ ~ *U*(0.01, 1) and *C* ~ *U*(100, 1500), which have been set according to values previously estimated by Wood et al. (2014). We sequentially implement the ABC algorithm by considering successively smaller tolerances, ϵ, to refine the parameter space. For each pair (λ, *C*) of parameters sampled from the priors, the birth-and-death process is simulated using the Gillespie algorithm to obtain the number of intracellular bacteria as predicted by the model (Gillespie, 2007). Once this number is predicted from our model, these values are compared with data by Lindemann et al. (2011). In particular, if *X*(*t*) is the amount of bacteria predicted by our within-phagocyte model at time *t*, and *Data*(*t*) is the number of bacteria observed at that time instant according to data by Lindemann et al. (2011), which are available for a set of time instants *T*, we make use of the Euclidean distance

(7) $$\begin{array}{l} d\left(Model\;Prediction,Data\right)\;=\;{\left(\underset{t\in T}{\sum }{\left(X\left(t\right)-Data\left(t\right)\right)}^{2}\right)}^{\frac{1}{2}}, \end{array}$$

so that each corresponding parameter pair (λ, *C*) is accepted only if *d*(*Model Prediction, Data*) < ϵ. At first the tolerance is set to ϵ = 100, so that an estimate of where the true parameters lie can be found. After this, the prior distributions are adjusted accordingly and the ABC algorithm is repeated for tolerances ϵ = 50, 25, 15, to converge around the posterior distribution (Toni et al., 2009). We note that threshold values ϵ = 100, 50, 25, 15 were chosen after a preliminary exploration of the parameter space and the corresponding distance measures between the model predictions and experimental measurements, so that a posterior sample of size 10^5^ could be obtained in around 48 h, by using the high performance computing ARC3 facilities at the University of Leeds. A bivariate histogram of the sample posterior distribution obtained in this way is provided in Figure 5, with the median of the sample indicated. Univariate histograms for each parameter are given on the corresponding axes.

### 3.2. Within-host model

Estimated parameter values α and μ for the within-host model proposed by Wood et al. (2014) were obtained using non-linear least squares to fit their within-host model to experimental data for the number of extracellular bacteria within the host during the initial 48 h post infection. Since our within-host model is part of a multi-scale model which incorporates a variable number of bacteria released on rupture of any infected phagocyte, new estimations for these parameter values are now required. Thus, ABC is used to calibrate the parameters (α, μ) of the within-host model depicted in Figure 3 by using within-host infection data (Eigelsbach et al., 1962; White et al., 1964). We note that this requires the distribution of the number of bacteria released on rupture. This has been described in the Supplementary Material, using the posterior median values of λ and *C* of Figure 5. This same rupture distribution is used in each iteration of the ABC algorithm at the within-host level. In keeping with Wood et al. (2014), and to represent the heterogeneities at the population level in individual susceptibility, *M* is not fixed and is considered instead a random value *M* ~ *logN*(26.2, 6.05), according to data by Saslaw et al. (1961) and Sawyer et al. (1966). These data report the amount of bacteria found within infected individuals at the time of symptoms onset. For small to moderate values of *M*, the exact analysis carried out in the Supplementary Material can be applied to compute the probability of response and the mean response time in the within-host model. On the other hand, stochastic simulation approaches need to be implemented for large values of *M*. We note that given the potential extremely large values of *M*, the Gillespie algorithm is not a viable choice to simulate the within-host infection dynamics for these values, and an approximate τ-leaping procedure is used instead, with adaptive step size (Cao et al., 2006).

Prior distributions assumed for each parameter are α ~ *U*(0, 1) and μ ~ *U*(0, 25). Because of the shorter intervals considered in the priors of these parameters compared to those in section 3.1, we carry out here a standard rejection ABC (i.e., not sequential) where 2 × 10^5^ iterations of the ABC algorithm were performed. Tolerance is set so that an acceptance rate of 1% is obtained, and a sample of size 2 × 10^3^ is obtained for the posterior distributions. Due to the large orders of magnitude for the number of extracellular bacteria within the host observed in the data by Eigelsbach et al. (1962) and White et al. (1964), we propose here to use the Euclidean distance as for (λ, *C*) but over the logarithm of the predicted values and the observed data by Eigelsbach et al. (1962) and White et al. (1964). That is, we consider the distance

(8) $$\begin{array}{l} d\left(Model\;Prediction,Data\right)\;=\;{\left(\underset{t\in T}{\sum }{\left(logX\left(t\right)-logData\left(t\right)\right)}^{2}\right)}^{\frac{1}{2}}. \end{array}$$

**Figure 6:**
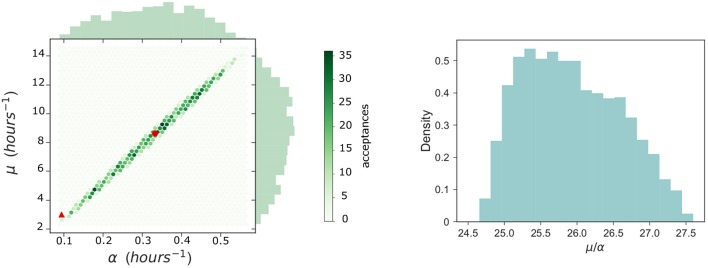
Within-host parameter estimation. **(Left)** A bivariate histogram for the parameters α and μ obtained as a result of the ABC procedure for the within-host model. The posterior median values are indicated with a red circle, while values (α, μ) = (0.0939, 3) *h*^−1^ estimated by Wood et al. (2014) are indicated with a red triangle. **(Right)** A posterior histogram for the ratio μ/α.

The results of the ABC lead to the posterior bivariate histogram of Figure 6, which clearly indicates a positive correlation between parameters α and μ, where most of the learning occurs about the ratio μ/α. We note that this positive correlation is directly related to the fact that, intuitively, α and μ rates correspond to within-host events which can be considered as *opposite* events in this system (one representing bacterial escape from the extracellular environment, facilitating disease, and the other representing bacterial death, preventing disease). Thus, our within-host model dynamics can replicate the experimental data by either considering that both events occur, simultaneously, at a slower or faster pace. However, we point out that since the (α, μ) joint distribution in Figure 6 (left) does not have the accepted sampled values homogeneously located all around the elliptic shape, where more accepted values can be found around the center of the ellipse than in the corners, one should consider that these parameter values (near the corresponding medians, given by the red circle) have larger posterior probability than the estimated values obtained by Wood et al. (2014) (red triangle). Final parameter values for the within-phagocyte and the within-host models are reported in Table 1.

**Table 1:** Parameter values for the within-phagocyte and within-host models.

**Parameter**	**Event**	**Parameter value**
λ	Intracellular bacterial replication	Estimated in Figure 5: 0.2164 *h*^−1^ (median)
*C*	Intracellular carrying capacity	Estimated in Figure 5: 393 *bacteria* (median)
μ	Extracellular bacterial death	Estimated in Figure 6: 8.63 *h*^−1^ (median)
α	Phagocytosis with bacterial survival	Estimated in Figure 6: 0.3325 *h*^−1^ (median)
*M*	Threshold value for symptoms onset	Randomly distributed *M* ~ *logN*(26.2, 6.05)
δ	Phagocyte rupture	δ = *Median*[*T*^*rupture*^]^−1^ = 0.0241 *h*^−1^
*R*_*k*_	Probability of rupture with *k* bacteria	From within-phagocyte model (Figure 10)

**Figure 7:**
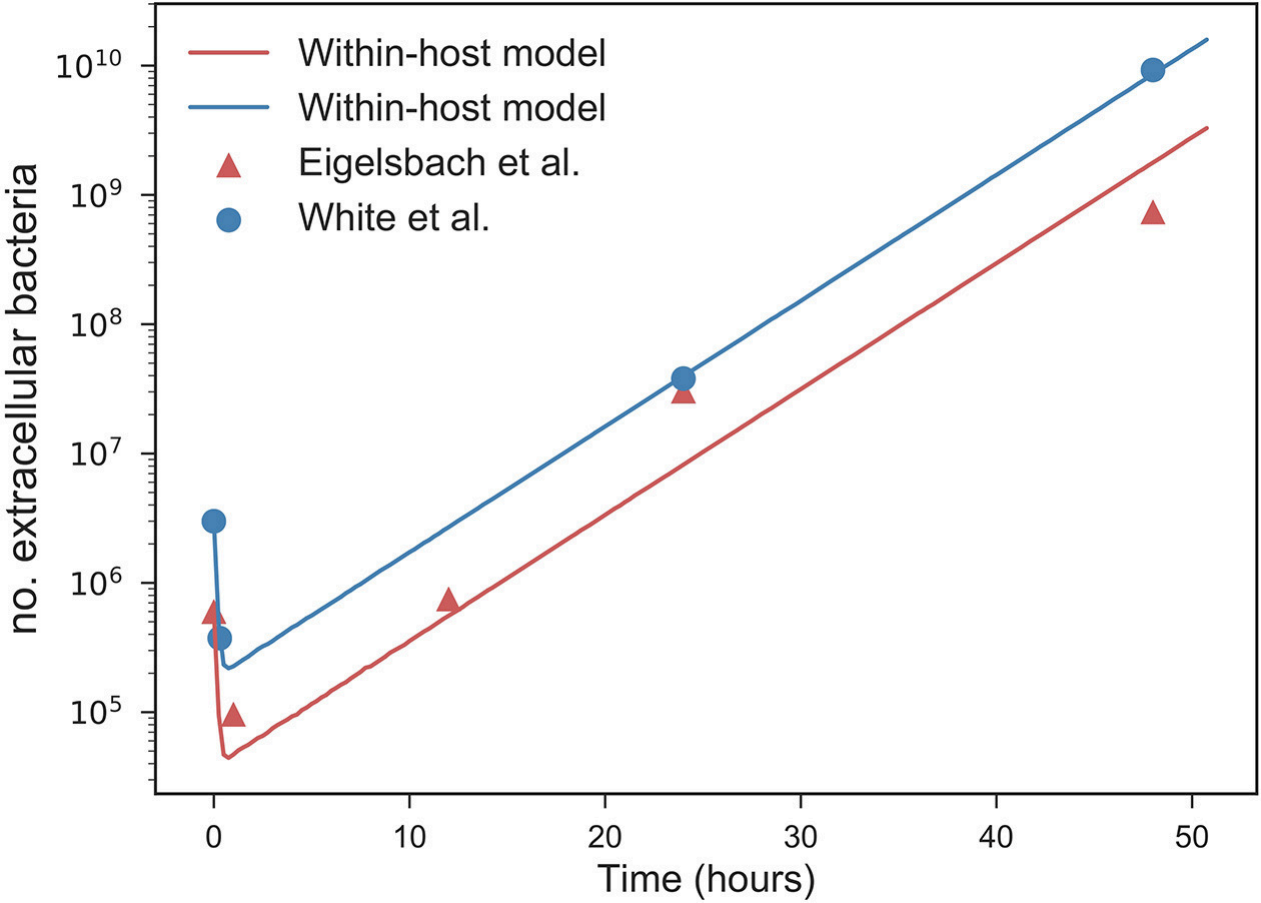
Predicted values of bacterial load by within-host model. Within-host model predictions (curves) obtained as mean values throughout time from Gillespie simulations for different initial bacterial loads (blue and orange) corresponding to the initial values measured by Eigelsbach et al. (1962) and White et al. (1964), vs. data points by Eigelsbach et al. (1962) and White et al. (1964). Median values of α and μ considered as computed in Figure 6.

We can compare our within-host model predictions, in terms of the number of bacteria throughout time, with the data by Eigelsbach et al. (1962) and White et al. (1964). In Figure 7 we plot the predictions made by our within-host model and compare them with the bacterial load data by Eigelsbach et al. (1962) and White et al. (1964), where the initial conditions are given as the corresponding data values at time *t* = 0. Similarly to results by Wood et al. (2014), our within-host model does better in predicting the data by White et al. (1964), where larger amounts of bacteria were measured within the host.

### 3.3. Population-level model

**Figure 8:**
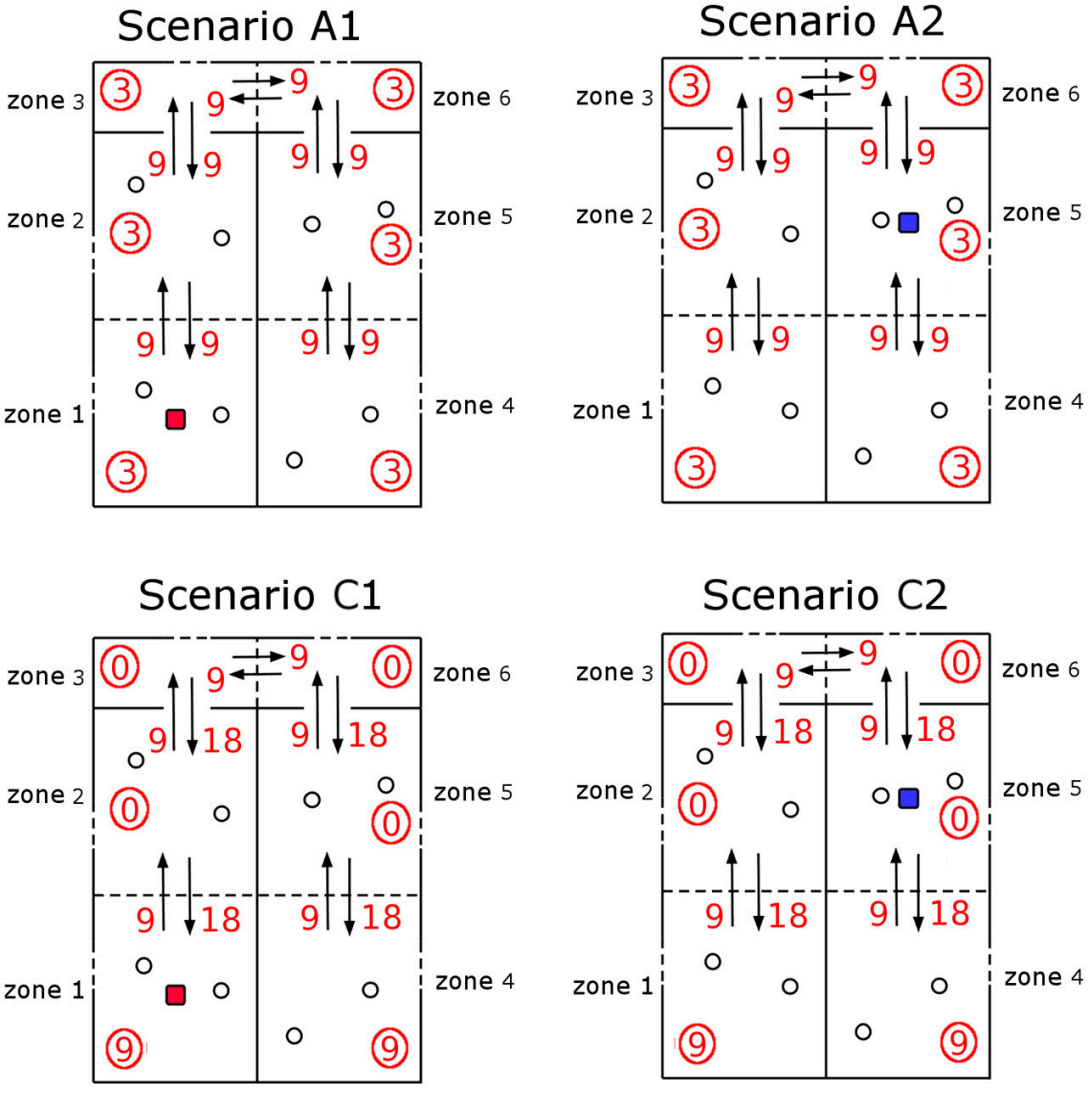
Ventilation scenarios considered in the microbiology laboratory. Four scenarios A1, A2, C1, and C2 corresponding to two potential release locations (zone 1, scenarios A1 and C1; zone 5, scenarios A2 and C2). Ventilation regime in scenarios A1 and A2 represents a well-mixed ventilation, where airflow (arrows, with β_*ik*_ rates given as red numbers) is well balanced across zones and same extract ventilation (circled values) is considered in all zones. Ventilation regime in scenarios C1 and C2 represents airflow occurring from the corridor areas to the opposed side of the rooms, where extract ventilation is in place.

**Figure 9:**
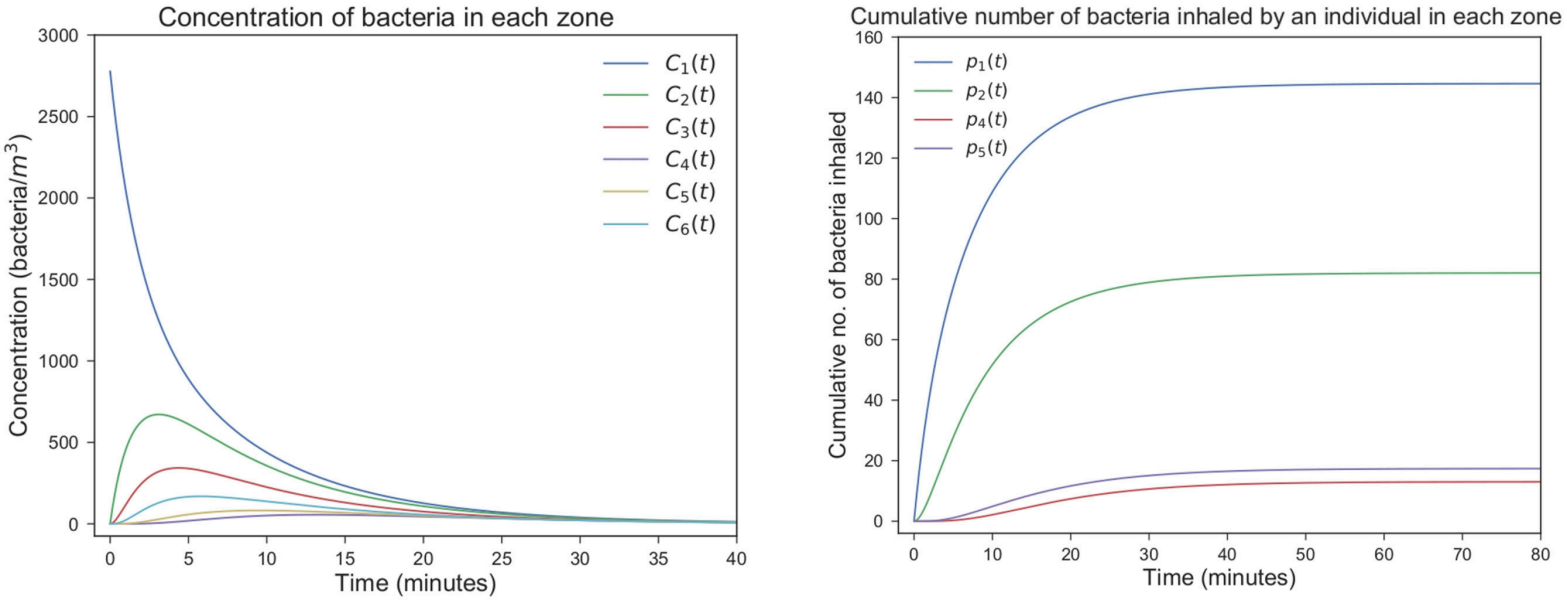
Predicted airborne spread and inhalation of bacteria in the laboratory. Time course of the variables *C*_*i*_(*t*), 1 ≤ *i* ≤ 6, and *p*_*j*_(*t*), *j* ∈ {1, 2, 4, 5}, for scenario *A*1.

Four different scenarios *A*1, *A*2, *C*1, and *C*2 are considered depending on two potential bacterial release locations (see Figure 4). Two potential ventilation regimes (A and C) within the microbiology laboratory have been chosen, as described in Table 2: ventilation regime A (scenarios A1 and A2) and ventilation regime C (scenarios C1 and C2) considered by Noakes and Sleigh (2009) and López-García et al. (under review). Regardless of the particular location where it occurs, it is assumed that 10^5^ bacterial counts are released at time *t* = 0. In each scenario it is assumed that $V_{i}=36m^{3}$ for *i* ∈ {1, 2, 4, 5} and $V_{i}=12m^{3}$ for *i* ∈ {3, 6}. The pulmonary rate is set to ρ = 0.01*m*^3^·*min*^−1^ (Noakes and Sleigh, 2009), while the remaining parameters in Figure 4 are provided in Table 2, along with the steady state values ${\text{p}}^{\left(k\right)}=\underset{t\to \infty }{\lim}\left(p_{1}^{\left(k\right)}\left(t\right),p_{2}^{\left(k\right)}\left(t\right),p_{4}^{\left(k\right)}\left(t\right),p_{5}^{\left(k\right)}\left(t\right)\right),k\in \left\{\text{A1, A2, C1, C2}\right\}$. A graphical representation of scenarios *A*1, *A*2, *C*1 and *C*2 is given in Figure 8, and the time course of the variables *C*_*i*_(*t*), 1 ≤ *i* ≤ 6, and *p*_*j*_(*t*), *j* ∈ {1, 2, 4, 5}, are plotted for scenario *A*1 in Figure 9 for illustrative purposes.

**Table 2:** Parameter values for four ventilation regimes.

**Scenario**	**$\beta_{ij}\left(m^{3}/\text{min}\right)$**	**$Q_{i}\left(m^{3}/\text{min}\right)$**	**Source room**	**Steady state**
*A*1	β_12_ = β_23_ = β_36_ = β_63_	*Q*_*i*_ = 3, *i* = 1, .., 6	1	***p***^(*A*1)^ = (145, 82, 13, 17)
	= β_56_ = β_45_ = β_21_ = β_32_		
	= β_65_ = β_54_ = 9			
*A*2	β_12_ = β_23_ = β_36_ = β_63_	*Q*_*i*_ = 3, *i* = 1, .., 6	5	***p***^(*A*2)^ = (17, 23, 82, 110)
	= β_56_ = β_45_ = β_21_ = β_32_		
	= β_65_ = β_54_ = 9			
*C*1	β_12_ = β_23_ = β_36_ = β_63_	*Q*_1_ = *Q*_4_ = 9	1	***p***^(*C*1)^ = (102, 46, 9, 9)
	= β_56_ = β_45_ = 9	*Q*_2_ = *Q*_3_		
	β_21_ = β_32_ = β_65_ = β_54_ = 18	= *Q*_5_ = *Q*_6_ = 0		
*C*2	β_12_ = β_23_ = β_36_ = β_63_	*Q*_1_ = *Q*_4_ = 9	5	***p***^(*C*2)^ = (18, 18, 92, 92)
	= β_56_ = β_45_ = 9	*Q*_2_ = *Q*_3_		
	β_21_ = β_32_ = β_65_ = β_54_ = 18	= *Q*_5_ = *Q*_6_ = 0		

*Airflow parameters for the four scenarios considered, and steady state bacterial intake values representing initial dose for individuals at each zone. Airflow parameters have been chosen according to those in the ventilation regimes A and C considered by Noakes and Sleigh (2009) and López-García et al. (under review)*.

## 4. Results

**Figure 10:**
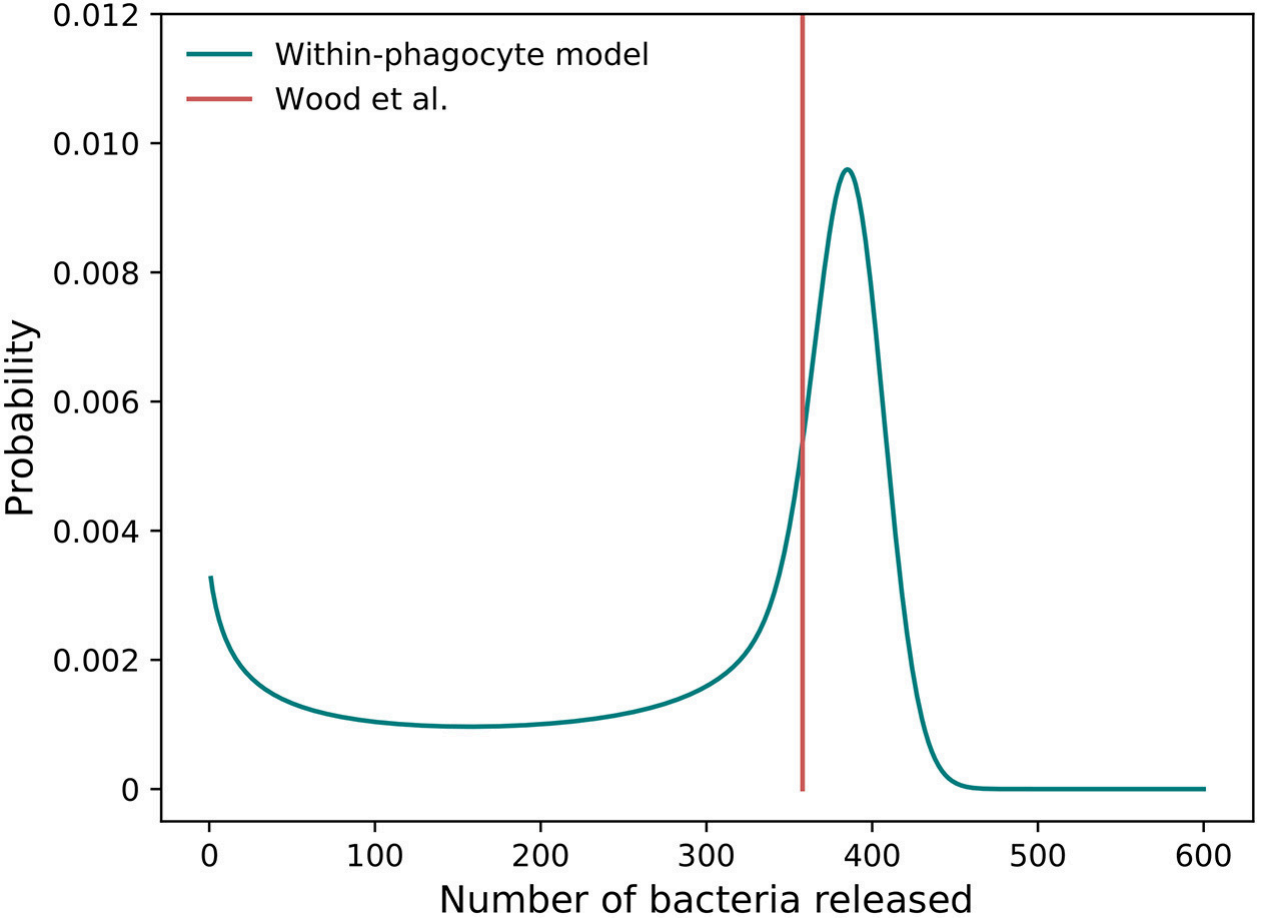
Predicted rupture size distribution. The distribution of the predicted number of bacteria released by a single phagocyte on rupture, as computed from the within-phagocyte model, compared to the fixed value assumed by Wood et al. (2014). The posterior median values for λ and *C* in Figure 5 are used to compute this distribution.

The distribution of the number of bacteria released by an infected phagocyte, for posterior median values of λ and *C* from Figure 5, is provided in Figure 10. In order to compare with results by Wood et al. (2014), let us note that the approach they use involves evaluating a deterministic logistic growth process at the median (log-normally distributed) time taken for an infected phagocyte to rupture. The method here may instead be interpreted as computing the distribution of the number of bacteria generated by means of the analogous stochastic logistic growth process, but when the actual log-normally distributed rupture time is incorporated into the model (see Figures 1B,C). Since the deterministic and stochastic processes have both been parameterized using the same data set, they are comparable, and the median number of bacteria released from our predicted distribution in Figure 10 is approximately equal to the fixed value of 358 bacteria released upon rupture estimated by Wood et al. (2014), supporting the fact that the median number of bacteria released had previously been estimated correctly. Despite this, the method outlined here is more general, since it allows to incorporate the log-normal distribution of rupture times, and thus, a more comprehensive analysis of the number of bacteria released can be conducted, and incorporated into the within-host dynamics, by considering inter-phagocyte rupture size variability. Moreover, we note that the mean number of bacteria released on rupture is predicted to be 288, significantly lower than the fixed value 358 considered by Wood et al. (2014). This is directly related to the bimodal shape of our predicted rupture size distribution, which suggests that some phagocytes will likely rupture with just a few bacteria, and that the total number of bacteria released by each single infected phagocyte was slightly over-estimated by Wood et al. (2014) on average. We note that our model is able to predict that a significant amount of phagocytes might rupture releasing few bacteria, which is something that the deterministic approach followed by Wood et al. (2014) does not reflect. We also note that the actual rupture size distribution, to the best of our knowledge, has not been experimentally measured *in vitro* yet, which would allow us to do model selection based on predictions in Figure 10. However, it has been recently experimentally observed by single-cell analysis (Brock and Parmely, 2017) that a significant amount of phagocytes can die releasing very few bacteria. While the deterministic amount of bacteria proposed by Wood et al. (2014) cannot account for this, our model predicts indeed a significant amount of phagocytes releasing very few bacteria, which is represented by the first mode in Figure 10. This suggests that this mode is not an artifact caused by the stochastic within-host model, but that phagocytes rupturing soon (according to the estimated log-normally distributed rupture time) would not have enough time for substantial bacterial proliferation, leading to small rupture sizes predicted by the model and being experimentally observed.

**Figure 11:**
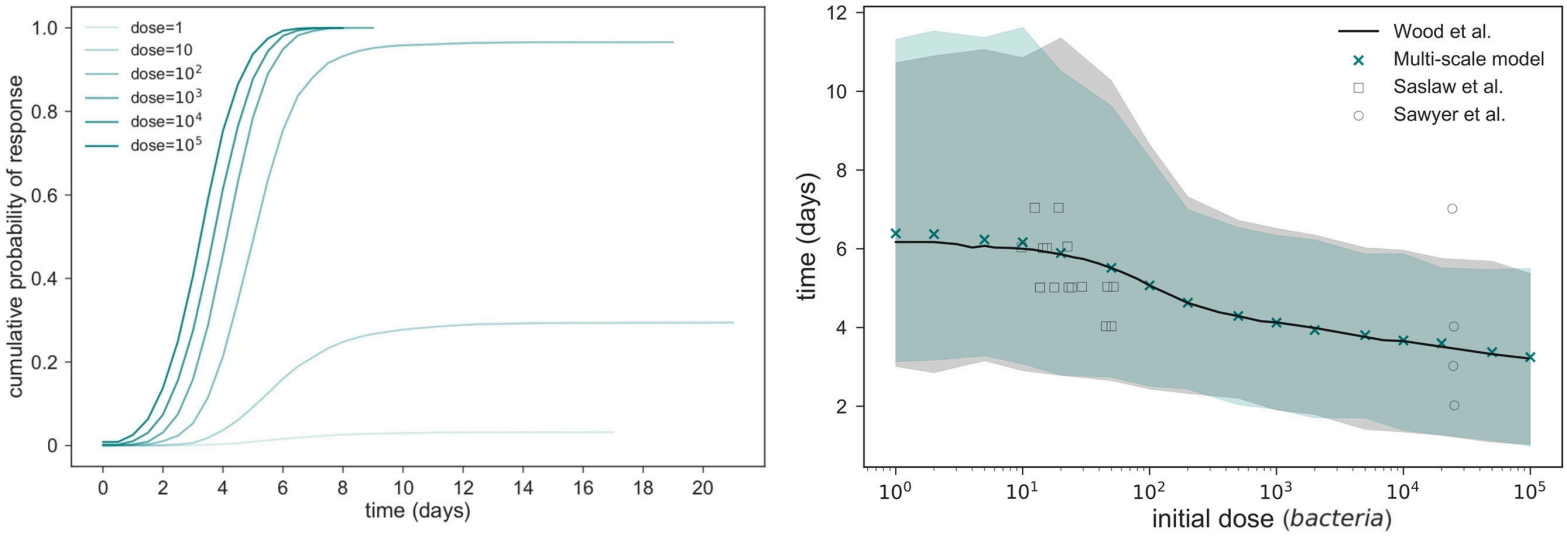
Within-host model predictions. **(Left)** Predicted cumulative probability of response up to time *t*, vs. *t* ≥ 0, from our multi-scale model and different initial doses. **(Right)** comparison between the conditioned mean time until response predicted by Wood et al. (2014) and by the multi-scale model developed here. Shaded regions represent 95% quantiles.

By using this rupture size distribution, and the within-host model in section 2.2, the probability of response and mean response times can be computed for varying initial doses. In Figure 11 (left), we plot the cumulative probability of response (i.e., cumulative probability of the process in Figure 3 reaching state *M*), as predicted from our model for different initial doses. We note that the asymptotic values in Figure 11 (left) represent the probabilities of response for each initial dose. We plot in Figure 11 (right) the (conditioned) mean time until response predicted for different initial doses, and compare this with the predictions by Wood et al. (2014) and with data of the time until symptoms onset observed in infected individuals (Saslaw et al., 1961; Sawyer et al., 1966). Our predictions are obtained by using the posterior median parameter values in Figures 5, 6. We note that, once parameters (α, μ) are estimated as explained in section 3.1, results obtained here for the probability of response and the (conditioned) mean response time are very similar to those previously found by Wood et al. (2014), indicating that the multi-scale model is not only capable of reproducing their results, but also corresponds well with the two experimental data sets by Saslaw et al. (1961) and Sawyer et al. (1966). However, we note that our multi-scale model only replicates well these results for posterior distribution of (α, μ) in Figure 6, where our predicted median values are far away from those parameters estimated by Wood et al. (2014). In particular, although these parameters are highly correlated and determining their individual true values is difficult, the histogram in Figure 6 suggests that the ratio of α and μ ranges from 24.69 to 27.54, which is lower than the ratio of 31.95 found by Wood et al. (2014). Moreover, our results in Figure 6 suggest that both α and μ were underestimated by Wood et al. (2014) (see the red circle and triangle in Figure 6).

**Figure 12:**
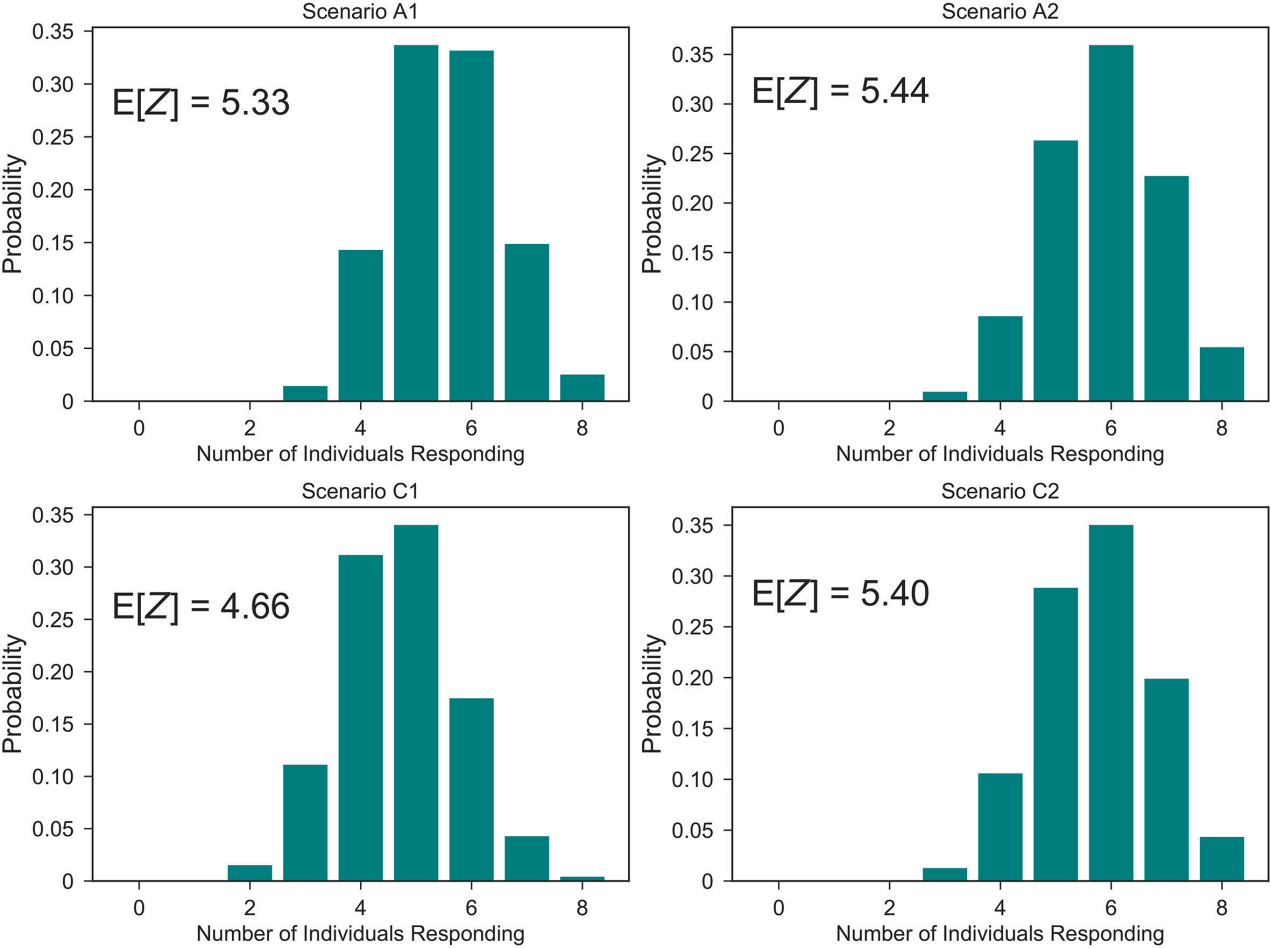
Predicted number of individuals showing symptoms in the laboratory. Distribution of the number *Z* of individuals suffering the infection upon bacterial release, out of the eight individuals in Figure 8, for scenarios *A*1, *A*2, *C*1, and *C*2. That is, probabilities ℙ(*Z* = *z*), 0 ≤ *z* ≤ 8.

At the population level, one can use the probability of response for each individual computed from the within-host model, where their initial dose is given by the steady state values in Table 2, in order to compute the distribution of the number *Z* of individuals within the laboratory showing symptoms after the bacterial release, for each of the four scenarios considered in Table 2. These distributions are plotted in Figure 12, together with the corresponding expected values *E*[*Z*]. From this, it can be seen that scenarios associated with smaller number of responses are *A*1 and *C*1, that is, when the bacteria are released from zone 1 as opposed to zone 5. This might be expected since air can flow from zone 5 into other areas more easily, whereas it only flows into one other zone from zone 1. However, an interplay between the ventilation regime (i.e., airflow dynamics) and the bacterial release location can be observed, where the ventilation regime in scenario *C*1 helps to decrease pathogen concentration in the release zone (zone 1), due to significant extract ventilation in place in this zone, while this same ventilation implies in scenario *C*2 the airborne spread of pathogen from zone 5 toward zone 4, causing more infections at the population level.

## 5. Discussion

In this work, we propose a multi-scale model for the infection dynamics of *F. tularensis* which covers the within-phagocyte, within-host and population scales. The within-host model should be considered an extension of the model originally proposed by Wood et al. (2014), where inter-phagocyte rupture size variability is incorporated in the distribution of the number of bacteria released upon rupture by any infected phagocyte. This distribution is computed by means of a stochastic logistic growth process for the replication of bacteria at the within-phagocyte level, but where the log-normally distributed rupture time predicted by Wood et al. (2014) is explicitly incorporated by means of a PH-type approximation. This approximation allows us to consider a Markovian stochastic process for the within-phagocyte infection dynamics. Once the extended within-host model is set up, we provide analytical approaches for computing the probability of response (in terms of the number of extracellular bacteria within the host to reach some response threshold *M*), and the mean time until this response takes place (conditioned on this response actually occurring). By calibrating the within-phagocyte and within-host model parameters using experimental infection data, our multi-scale model predictions are in agreement with experimental data both at the within-phagocyte and within-host level.

The main advantages of our multi-scale model are:

- The within-phagocyte model allows us to incorporate the estimated log-normally distributed rupture time into the bacterial proliferation dynamics, while keeping the Markovian nature of the original process. This allows the exact distribution of the rupture size to be computed. We believe that our methodology, using phase-type approximations for incorporating non-Markovian events in these intracellular processes, as well as the first-step arguments considered here for computing the rupture size distribution, is applicable to other intracellular bacterial replication systems.
- The rupture size distribution computed by our model and plotted in Figure 10 is able to capture the fact that a significant amount of phagocytes might die releasing very few bacteria, which has been recently experimentally observed (Brock and Parmely, 2017).
- The stochastic nature of the within-phagocyte model incorporates inter-phagocyte variability in the rupture size in the within-host model, relaxing the assumption made by Wood et al. (2014) that every phagocyte releases a fixed amount of bacteria. Relaxing this assumption leads to different predictions in the posterior estimated values of within-host parameters (α, μ), as shown in Figure 6, with respect to previous predictions made by Wood et al. (2014). This is directly related to the fact that different behaviors can be expected when the within-host model is simulated with the actual rupture distribution (so that each phagocyte, upon rupture, can release different numbers of bacteria with different probabilities) instead of considering that every phagocyte releases a fixed number of bacteria, even if this fixed release is set equal to the median value of the distribution in Figure 10.
- The zonal ventilation model is a simple but flexible way of representing airborne spread of bacteria, and of linking this spread with the initial doses infecting each of the individuals in the laboratory under study. Our results suggest that there is a clear interplay between the potential release location and the ventilation in place within the laboratory, where an appropriate ventilation regime can decrease the number of individuals developing symptoms.

The original model by Wood et al. (2014), as well as the extended model proposed here, should be considered as one of the few and recent attempts to propose mechanistic models for the computation of dose-response probabilities and the mean time until individuals showing symptoms following bacterial infection. Many of the original approaches in the literature to this aim usually involve adjusting exponential and beta-Poisson models to data (Chen, 2007; Huang and Haas, 2009). These models are limited since the real within-host biological mechanisms at play are not explicitly considered, and the distributions are selected only due to their ability to approximate the experimental or clinical data. Moreover, timescales for the different within-host processes are usually not explicitly considered in these models, where the final output of the model is usually limited to the dose-response probability curve. Thus, recent attempts are being made in order to explicitly consider the biological mechanisms following bacterial infection, leading to computational models which can analyse the timescales of these intracellular and within-host processes, not only for *F. tularensis* but also for other pathogens such as anthrax (Day et al., 2011).

Developing new mathematical and computational models that can explicitly account for biological mechanisms requires a significant amount of quantitative experimental data, and a balance between model complexity and experimental information must always be struck. For example, in our within-host model, all the mechanisms leading to extracellular bacterial death, such as the complement system, antibodies, natural killer cells, antimicrobial peptides or phagocytosis leading to bacterial killing are represented as a single event occurring at rate μ. If one were to distinguish all of these events in the model, experimental measurements of the specific contribution of each mechanism would be required, and a new version of our multi-scale model could be proposed. An additional limitation of our model, at the within-phagocyte level, is the fact that the rupture time is modeled as a log-normally distributed time which is independent of the bacterial proliferation dynamics simultaneously occurring within the phagocyte. Ideally, if we had enough experimental knowledge about the effect that the bacterial load has on the rupture of the phagocyte, one could consider that the rate of rupture from any state *n* in Figure 1 (i.e., *n* bacteria within the phagocyte at a given time) is a function δ_*n*_ of this bacterial load. Thus, using the independent log-normally distributed time estimated by Wood et al. (2014) should be seen as a compromise between current experimental knowledge and model complexity, and is based on the fact that bacterial escape into the cytosol has been shown to be both essential and sufficient for triggering caspase-3 activation, which is the mechanism thought to induce cell death (Santic et al., 2010). This also agrees well with recent experimental evidence (Brock and Parmely, 2017) showing that cell death does not require high bacterial burden, nor does a large number of intracellular bacteria ensure immediate phagocyte rupture. Finally, at the population-level, we note that more elaborated fluid dynamics simulations could be considered for the airborne spread of *F. tularensis* in the microbiology laboratory. We propose here a zonal ventilation model as a simple but flexible way of linking the indoor airflow dynamics with the initial dose of each individual after a bacterial release. We note however that the imprecisions inherently caused by the spatial discretisation in this zonal ventilation approach, where the indoor setting is split in a number of zones and the air is assumed to be well-mixed within each zone, can be reduced by increasing the number of zones under consideration.

The development of a mathematical model of infection dynamics at different scales is a challenging problem for which few successful attempts have been made in the literature so far (Bauer et al., 2009). To the best of our knowledge, this is the first multi-scale model for *F. tularensis* trying to account for the infection dynamics from the intracellular to the population level. It is conceivable that the future of *in silico* modeling will consist of a large number of interconnected models at different scales, and where one of the main aims will be to predict the effects that perturbations of model parameters along the different scales can have in the global infection dynamics. Finally, the approach presented in this article could also be readily applied to investigate the potential casualty impacts resulting from a deliberate bioterrorism or biological warfare attack in civilian and military scenarios. For instance, our multi-scale model may be used in conjunction with a larger-scale outdoor dispersion model that produces *F. tularensis* concentration estimates over large areas of terrain.

## Author contributions

All authors conceived the idea, contributed to develop the mathematical models, wrote and reviewed the manuscript. JC and ML-G carried out the analysis of the stochastic descriptors for all the three models, and the Bayesian analysis for estimating parameter values from data. JC developed the numerical codes and prepared all the figures. All authors were involved in the writing of the manuscript.

## Data statement

Computer codes (in *Python*) for reproducing our results are available at (Carruthers et al., 2018).

### Conflict of interest statement

The authors declare that the research was conducted in the absence of any commercial or financial relationships that could be construed as a potential conflict of interest.
