# Supplementary material for: A Novel Stochastic Multi-Scale Model of Francisella tularensis Infection to Predict Risk of Infection in a Laboratory
Source: Front Microbiol. 2018 Jul 6;9:1165. doi: 10.3389/fmicb.2018.01165 (PMC6043654; doi:10.3389/fmicb.2018.01165)
Supplement: Supplementary file 1 [file Data_Sheet_1.pdf]

Supplementary Material: A novel stochastic multi-scale model of *Francisella tularensis* infection to predict risk of infection in a laboratory

1 ANALYSIS OF THE WITHIN-PHAGOCYTE MODEL

1.1 PH approximation of the log-normally distributed rupture time

Process in (Carruthers et al., 2018, Figure 1b)) contains an inter-event time which is log-normally distributed (representing the time until phagocyte rupture and bacterial release), which makes the process non-Markovian. In order to keep the Markovian nature of the process, we propose in Carruthers et al. (2018) to approximate the log-normal distribution for the rupture time with a $PH(\boldsymbol{\eta}, \mathbf{T})$ distribution, since the family of PH-type distributions is dense within the family of non-negative continuous distributions (He (2014)).

We note here that a $PH(\boldsymbol{\eta}, \mathbf{T})$ distribution can be defined as the absorption time for an absorbing continuous-time Markov chain (CTMC) $\mathcal{W}$. Given an absorbing CTMC $\mathcal{W}$ over the space of states $\mathcal{S}_{\mathcal{W}} = \{1, 2, \dots, m\} \cup \{B\}$, with B the absorbing state, one can express its infinitesimal generator as

$$\mathbf{Q} = \begin{pmatrix} \mathbf{T} & \mathbf{t} \\ \mathbf{0} & 0 \end{pmatrix},$$

where matrix $\mathbf{T}$ contains the transition rates of the Markov chain for states in $\{1, \dots, m\}$, and vector $\mathbf{t}$ contains those transition rates from states in $\{1, \dots, m\}$ to the absorbing state B. Thus, if one defines T as the time until absorption for process $\mathcal{W}$ (*i.e.,* the time until process $\mathcal{W}$ reaches state B), we say that $T \sim PH(\eta, \mathbf{T})$, where $\mathbf{T}$ is the sub-matrix of the infinitesimal generator represented above and $\boldsymbol{\eta}$ is the vector representing the initial distribution of the CTMC $\mathcal{W}$ over $\mathcal{S}_{\mathcal{W}}$.

In order to fully analyse the within-phagocyte model depicted in (Carruthers et al., 2018, Figure 1b)), the log-normal distribution, that determines when the rupture event occurs, is first approximated using a PH distribution. To do this, an auxiliary Markov process $\mathcal{W} = \{W(t) : t \geq 0\}$ with state space $\mathcal{S}_{\mathcal{W}} = \{\mathrm{I}, \mathrm{II}, ..., Z\} \cup \{B\}$ can be constructed such that the time taken for $\mathcal{W}$ to reach B is approximately $logN(3.72, 0.385)$ distributed. Here, B is the same absorbing state as in the process $\mathcal{X}$ in (Carruthers et al., 2018, Figure 1b)-c)), and Z denotes the number of transient states in the state space of $\mathcal{W}$, which is a parameter that can be chosen during the PH approximation procedure. Thus, process $\mathcal{W}$ can be thought of as a *clock* for the rupture event to occur, so that as $\mathcal{W}$ transitions across states in $\mathcal{S}_{\mathcal{W}}$, the rupture event becomes closer.

Let $q_{i,j}$ denote the transition rate from state i to state j for process $\mathcal{W}$. It is then possible to find Z and rates $\{q_{i,j} : i, j \in \mathcal{S}_{\mathcal{W}}\}$ such that the time taken for $\mathcal{W}$ to be absorbed approximately follows the desired distribution $logN(3.72, 0.385)$. In particular, a moment matching algorithm is applied here to do this and is implemented using the statistical software R (Osogami and Harchol-Balter (2006)). The PH distribution

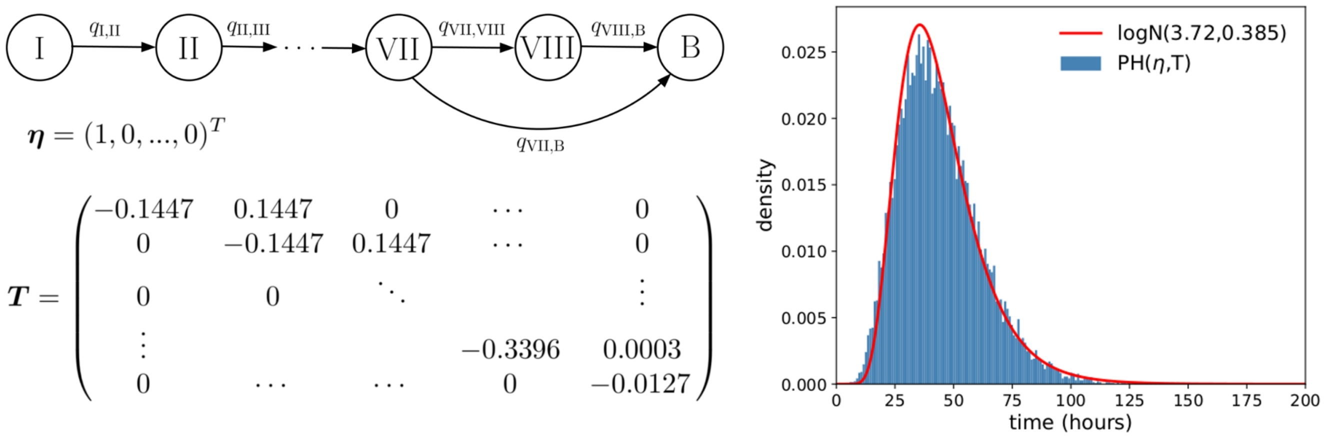

Figure S1. Left: A depiction of the one-dimensional Markov process $\mathcal{W}$ associated with the $PH(\boldsymbol{\eta}, \mathbf{T})$ distribution considered, so that the time to reach state B approximately follows $T^{rupture} \stackrel{\sim}{\sim} logN(3.72, 0.385)$. **Right**: Plot showing how accurately the $PH(\boldsymbol{\eta}, \mathbf{T})$ distribution approximates the desired log-normal distribution

is parametrised by a vector of initial probabilities containing the probability that $\mathcal{W}$ starts in each state of $\mathcal{S}_{\mathcal{W}}$, $\boldsymbol{\eta}$, and a matrix of transition rates between the transient states of $\mathcal{S}_{\mathcal{W}}$, $\mathbf{T}$. We plot in Figure S1 the structure of the process $\mathcal{W}$ obtained, with parameters $\boldsymbol{\eta}$ and $\mathbf{T}$ of the corresponding PH distribution, and provide a plot to depict how accurately the log-normal distribution is being approximated. In particular, this approximative $PH(\boldsymbol{\eta}, \mathbf{T})$ distribution is defined over the space of states $\mathcal{S}_{\mathcal{W}} = \{I, II, \ldots, VIII\} \cup \{B\}$, with vector $\boldsymbol{\eta} = (1, 0, \ldots, 0)^T$ representing that the absobring Markov process $\mathcal{W}$ (*i.e.,* the rupture time clock) starts at state I with probability 1, and matrix $\mathbf{T}$ given by

$$\mathbf{T} \quad = \quad \begin{pmatrix} -q_{I,II} & q_{I,II} & 0 & 0 & 0 & 0 & 0 & 0 \\ 0 & -q_{II,III} & q_{II,III} & 0 & 0 & 0 & 0 & 0 \\ 0 & 0 & -q_{III,IV} & q_{III,IV} & 0 & 0 & 0 & 0 \\ 0 & 0 & 0 & -q_{IV,V} & q_{IV,V} & 0 & 0 & 0 \\ 0 & 0 & 0 & 0 & -q_{V,VI} & q_{V,VI} & 0 & 0 \\ 0 & 0 & 0 & 0 & 0 & -q_{VI,VII} & q_{VI,VII} & 0 \\ 0 & 0 & 0 & 0 & 0 & 0 & -q_{VII,VIII} - q_{VII,B} & q_{VII,VIII} \\ 0 & 0 & 0 & 0 & 0 & 0 & 0 & -q_{VIII,B} \end{pmatrix}$$

$$= \begin{pmatrix} -0.1447 & 0.1447 & 0 & 0 & 0 & 0 & 0 & 0 \\ 0 & -0.1447 & 0.1447 & 0 & 0 & 0 & 0 & 0 \\ 0 & 0 & -0.1447 & 0.1447 & 0 & 0 & 0 & 0 \\ 0 & 0 & 0 & -0.1447 & 0.1447 & 0 & 0 & 0 \\ 0 & 0 & 0 & 0 & -0.1447 & 0.1447 & 0 & 0 \\ 0 & 0 & 0 & 0 & 0 & -0.1447 & 0.1447 & 0 \\ 0 & 0 & 0 & 0 & 0 & 0 & -0.3396 & 0.0003 \\ 0 & 0 & 0 & 0 & 0 & 0 & 0 & -0.0127 \end{pmatrix}.$$

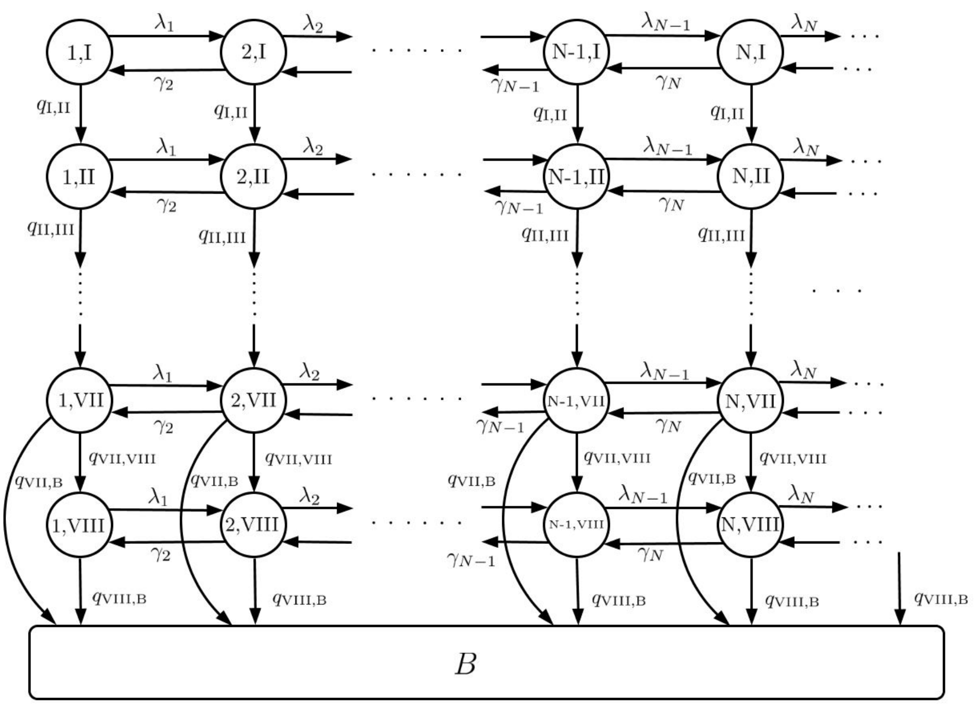

Figure S2. Diagram representing the within-phagocyte process $\mathcal{X}$ in (Carruthers et al., 2018, Figure 1c)), where the approximative PH distribution can be seen as a *clock* for the rupture event

Once this log-normal distribution is replaced by the PH approximative distribution, the within-phagocyte process in (Carruthers et al., 2018, Figure 1b)) becomes the process in (Carruthers et al., 2018, Figure 1c)). By using the PH diagram representation in Figure S1, the within-phagocyte process in (Carruthers et al., 2018, Figure 1c)) can be represented as in Figure S2. Each state (i, j) of the process in Figure S2 represents i intracellular bacteria at a particular time instant, where the rupture clock is at state $j \in \{I, \dots, VIII\}$. Thus, arrows going downwards in Figure S2 represent the rupture clock moving towards the rupture event, while right-left arrows represent the logistic growth process which is occurring at the same time for the replication of bacteria. Once this second component j reaches the absorbing state B (which occurs after a PH distributed time which approximates the $logN(3.72, 0.385)$ distributed rupture time), the number of bacteria released is given by the state i when this occurs.

Since $\boldsymbol{\eta} = (1, 0, \dots, 0)^T$, one would set the rupture event clock into state I at time $t = 0$. However, when *F. tularensis* bacteria first enters a host phagocyte, there is a period of approximately one hour where the bacteria is contained within a phagosome and does not replicate (Golovliov et al. (2003)). This is not currently accounted for in the within-phagocyte model since replication of bacteria is allowed to begin from $t = 0$. To account for this phagosomal stage, the assumption is made that during the first hour, since the clock on the time to rupture has already begun, the process $\mathcal{X}$ in Figure S2 would only be allowed to transition between states in this clock phase. That is, if the process in Figure S2 starts at state $(i, j) = (1, I)$ at time $t = 0$, only component j is allowed to progress during the first hour, since there is no bacterial replication during this time. This can be easily incorporated into our model by considering the initial time $t = 0$ as the time instant at $1 - hour$ post infection. Thus, instead of considering that the rupture clock starts at I at time $t = 0$ (*i.e.,* instead of considering $\boldsymbol{\eta} = (1, 0, \dots, 0)^T$), one would consider that this rupture clock has already been working for one hour. To implement this, the transition probability matrix is first constructed for the auxiliary process $\mathcal{W}$. From this, the probability that $\mathcal{W}$ is in each of states $\{I, II, \dots, VIII, B\}$ after one hour can be computed. Once these probabilities have been computed, they

are stored in an updated vector $\boldsymbol{\eta}$, which is the initial vector to be considered at time $t = 0$ for process in Figure S2.

1.2 Truncation of the space of states

We carry out here a stochastic analysis of the within-phagocyte model in (Carruthers et al., 2018, Figure 1c)), depicted in Figure S2. In particular, the main aim in this within-phagocyte model is to compute the rupture size distribution (*i.e.,* the number of bacteria released by any infected phagocyte), which is given by the number of bacteria i once arrival to state B (*i.e.,* phagocyte rupture) occurs. In order to do this, although an infinite possible number of intracellular bacteria $\mathbb{N} = \{1, 2, 3, \dots\}$ is allowed in the model, it is clear that a physical carrying capacity exists for the number of bacteria that a single infected phagocyte can contain, which would lead us to consider a truncated set of states $\{(i, j) : \ i \in \{1, 2, 3, \dots, N\}, \ j \in \{I, \dots, VIII\}\} \cup \{B\}$ in Figure S2. In order to choose this truncating value N, we propose to set it so that discarded states $\{(i, j) : \ i \in \{N+1, N+2, \dots\}, \ j \in \{I, \dots, VIII\}\}$ are only visited with probability smaller than some small value ϵ, so that removing these states does not significantly affect the dynamics of the underlying stochastic process (Gómez-Corral and López-García (2015)).

Let $\alpha^N_{(i,j)}$ denote the probability that given the initial state $(i, j) \in \{(i, j) : \ i \in \{1, 2, 3, \dots, N\}, \ j \in \{I, \dots, VIII\}\}$, component i in process $\mathcal{X}$ reaches value $N + 1$ before being absorbed into state B. If we store these probabilities in vectors $\boldsymbol{\alpha}^N_i = \left(\alpha^N_{(i,\mathrm{I})}, \dots, \alpha^N_{(i,\mathrm{VIII})}\right)^T$, for $1 \leq i \leq N$, and if states in $\{(i, j) : \ i \in \{1, 2, 3, \dots, N\}, \ j \in \{I, \dots, VIII\}\}$ are organised by levels as

$$L(i) \quad = \quad \{(i, j) : \ j \in \{I, \dots, VIII\}\}, \quad 1 \leq i \leq N,$$

and levels are organised as $L(1) \prec L(2) \prec \cdots \prec L(N)$, one can obtain by means of first-step analysis the following matrix equation:

$$\begin{pmatrix} \boldsymbol{\alpha}^N_1 \\ \boldsymbol{\alpha}^N_2 \\ \vdots \\ \boldsymbol{\alpha}^N_{N-1} \\ \boldsymbol{\alpha}^N_N \end{pmatrix} = \begin{pmatrix} \mathbf{A}_{11} & \mathbf{A}_{12} & & & \\ \mathbf{A}_{21} & \mathbf{A}_{22} & \mathbf{A}_{23} & & \\ & \ddots & \ddots & \ddots & \\ & & \mathbf{A}_{N-1,N-2} & \mathbf{A}_{N-1,N-1} & \mathbf{A}_{N-1,N} \\ & & & \mathbf{A}_{N,N-1} & \mathbf{A}_{N,N} \end{pmatrix} \begin{pmatrix} \boldsymbol{\alpha}^N_1 \\ \boldsymbol{\alpha}^N_2 \\ \vdots \\ \boldsymbol{\alpha}^N_{N-1} \\ \boldsymbol{\alpha}^N_N \end{pmatrix} + \begin{pmatrix} \mathbf{0} \\ \mathbf{0} \\ \vdots \\ \mathbf{0} \\ \mathbf{A}_{N,N+1}\mathbf{1}_8 \end{pmatrix}$$

where $\mathbf{1}_8$ is a column vector with eight ones, and matrices $\mathbf{A}_{i,i-1}$, $\mathbf{A}_{i,i}$ and $\mathbf{A}_{i,i+1}$ each have dimension (8×8) and contain the respective probabilities that $\mathcal{X}$ transitions from states in level $L(i)$ to states in levels

$L(i-1)$, $L(i)$ or $L(i+1)$, respectively. Expressions for these matrices are

$$(\mathbf{A}_{i,i-1})_{j,j} = \gamma_i \left(\lambda_i + \gamma_i + \sum_{\substack{j'=I \\ j' \neq j}}^{VIII} q_{j,j'} + q_{j,B} \right)^{-1}, \quad i = 2, ..., N, \; j = I, ..., VIII \, ,$$

$$(\mathbf{A}_{i,i})_{j,l} = q_{j,l} \left(\lambda_i + \gamma_i + \sum_{\substack{j'=I \\ j' \neq j}}^{VIII} q_{j,j'} + q_{j,B} \right)^{-1}, \quad i = 1, ..., N, \; j,l = I, ..., VIII \, ,$$

$$(\mathbf{A}_{i,i+1})_{j,j} = \lambda_i \left(\lambda_i + \gamma_i + \sum_{\substack{j'=I \\ j' \neq j}}^{VIII} q_{j,j'} + q_{j,B} \right)^{-1}, \quad i = 1, ..., N, \; j = I, ..., VIII \, .$$

The solution to the above matrix equation is calculated recursively using Algorithm 1. Once these probabilities are in hand, we set in our numerical results in Carruthers et al. (2018) the value of N so that $\alpha^N_{(1,\sim\boldsymbol{\eta})} < 10^{-5}$; that is, if the rupture clock starts according to distribution $\boldsymbol{\eta}$, and the phagocyte has 1 single bacteria at this initial time $t = 0$, we set N so that the probability of process $\mathcal{X}$ exceeding value N is less than 10^{-5}.

Algorithm 1: Computation of the probability of process $\mathcal{X}$ reaching $N + 1$ bacteria before rupture

Step 1: $\mathbf{H}_1 = \mathbf{I}_8 - \mathbf{A}_{1,1}$;
  For $n = 2, ..., N$:
    $\mathbf{H}_n = \mathbf{I}_8 - \mathbf{A}_{n,n-1}\mathbf{H}^{-1}_{n-1}\mathbf{A}_{n-1,n} - \mathbf{A}_{n,n}$;
Step 2: $\boldsymbol{\alpha}^N_N = \mathbf{H}^{-1}_N \mathbf{A}_{N,N+1}\mathbf{1}_8$;
  For $n = N - 1, ..., 1$:
    $\boldsymbol{\alpha}^N_n = \mathbf{H}^{-1}_n \mathbf{A}_{n,n+1}\boldsymbol{\alpha}^N_{n+1}$.

1.3 Rupture size distribution

Let $R^{(k)}_{(i,j)}$ (denoted as R_k in Carruthers et al. (2018), where the initial state (i, j) is omitted for the ease of notation) denote the probability that $\mathcal{X}$ enters state B from one of states in $\{(k, \mathrm{I}), (k, \mathrm{II}), ..., (k, \mathrm{VIII})\}$, provided that it starts in state (i, j); that is, the probability that the phagocyte releases k bacteria upon rupture. We note that for results in Carruthers et al. (2018), we consider initial state $(1, j \sim \boldsymbol{\eta})$ where $i = 1$ represents that each macrophage is infected by a single bacterium, and j is chosen according to the initial distribution $\boldsymbol{\eta}$ described above. By conditioning on the subsequent state of $\mathcal{X}$, first-step arguments can be used to obtain the following expression for $R^{(k)}_{(i,j)}$:

$$R_{(i,j)}^{(k)} = \frac{1}{\lambda_i + \gamma_i + \sum\limits_{\substack{j'=I \\ j' \neq j}}^{VIII} q_{j,j'} + q_{j,B}} \left[\lambda_i R_{(i+1,j)}^{(k)} + \gamma_i R_{(i-1,j)}^{(k)} + \sum_{\substack{j'=I \\ j' \neq j}}^{VIII} q_{j,j'} R_{(i,j')}^{(k)} + q_{j,B} 1_{i=k} \right],$$

for $i \in \{1, ..., N\}$, $j \in \{\text{I}, ..., \text{VIII}\}$. This scalar expression may be written in matrix form as follows:

$$\begin{pmatrix} \mathbf{R}_1^{(k)} \\ \mathbf{R}_2^{(k)} \\ \vdots \\ \mathbf{R}_{N-1}^{(k)} \\ \mathbf{R}_N^{(k)} \end{pmatrix} = \begin{pmatrix} \mathbf{A}_{11} & \mathbf{A}_{12} & & & \\ \mathbf{A}_{21} & \mathbf{A}_{22} & \mathbf{A}_{23} & & \\ & \ddots & \ddots & \ddots & \\ & & \mathbf{A}_{N-1,N-2} & \mathbf{A}_{N-1,N-1} & \mathbf{A}_{N-1,N} \\ & & & \mathbf{A}_{N,N-1} & \mathbf{A}_{N,N} \end{pmatrix} \begin{pmatrix} \mathbf{R}_1^{(k)} \\ \mathbf{R}_2^{(k)} \\ \vdots \\ \mathbf{R}_{N-1}^{(k)} \\ \mathbf{R}_N^{(k)} \end{pmatrix} + \begin{pmatrix} \mathbf{0} \\ \mathbf{0} \\ \vdots \\ \mathbf{b}_k \\ \vdots \\ \mathbf{0} \end{pmatrix}, \qquad \text{(S1)}$$

where $\mathbf{R}_i^{(k)} = \left(R_{(i,I)}^{(k)}, ..., R_{(i,VIII)}^{(k)} \right)^T$, and where the (8×1) column vector $\mathbf{b}_k$ is given by:

$$(\mathbf{b}_k)_j = q_{j,B} \left(\lambda_k + \gamma_k + \sum_{\substack{j'=I \\ j' \neq j}}^{VIII} q_{j,j'} + q_{j,B} \right)^{-1}, \qquad j \in \{I, ..., VIII\}.$$

Finally, an adapted version of Algorithm 1, not reported here, allows to efficiently compute probabilities $R_{(i,j)}^{(k)}$ from Eq. (S1).

1.4 Rupture size distribution with additional phagocyte infection events

In our work, we have assumed in Section 2.1, that each phagocyte is infected by a single bacterium, so that the process in Figure 1 leads to the rupture size distribution represented in Figure 9. This is not only for our results to be comparable to those by Wood et al. (2014), where the same assumption was made, but also is based on experimental evidence by Golovliov et al. (2003), which suggests that during the initial phase of the infection, on average only one or two intracellular bacteria per phagocyte were observed. In order to explore the impact of this assumption, we compute here the analogous rupture size distribution to that in Figure 9, but when several extracellular bacteria are allowed to enter into the phagocyte until phagocyte rupture occurs. In particular, we consider that, after initial phagocyte infection by a single bacterium ($X(0) = 1$ in Section 2.1), up to n bacteria are allowed to enter into the phagocyte during the replication process and until phagocyte rupture, and that these additional infecting bacteria carry out the same intracellular stochastic logistic growth process described in Section 2.1. These n additional bacterial intake events occur at time instants uniformly distributed in $[0, Median[T^{rupture}]]$, with $Median[T^{rupture}] = 41.5\ hours$. Intake events occurring after $T^{rupture}$ are discarded, since they would take place after the phagocyte rupture actually occurs.

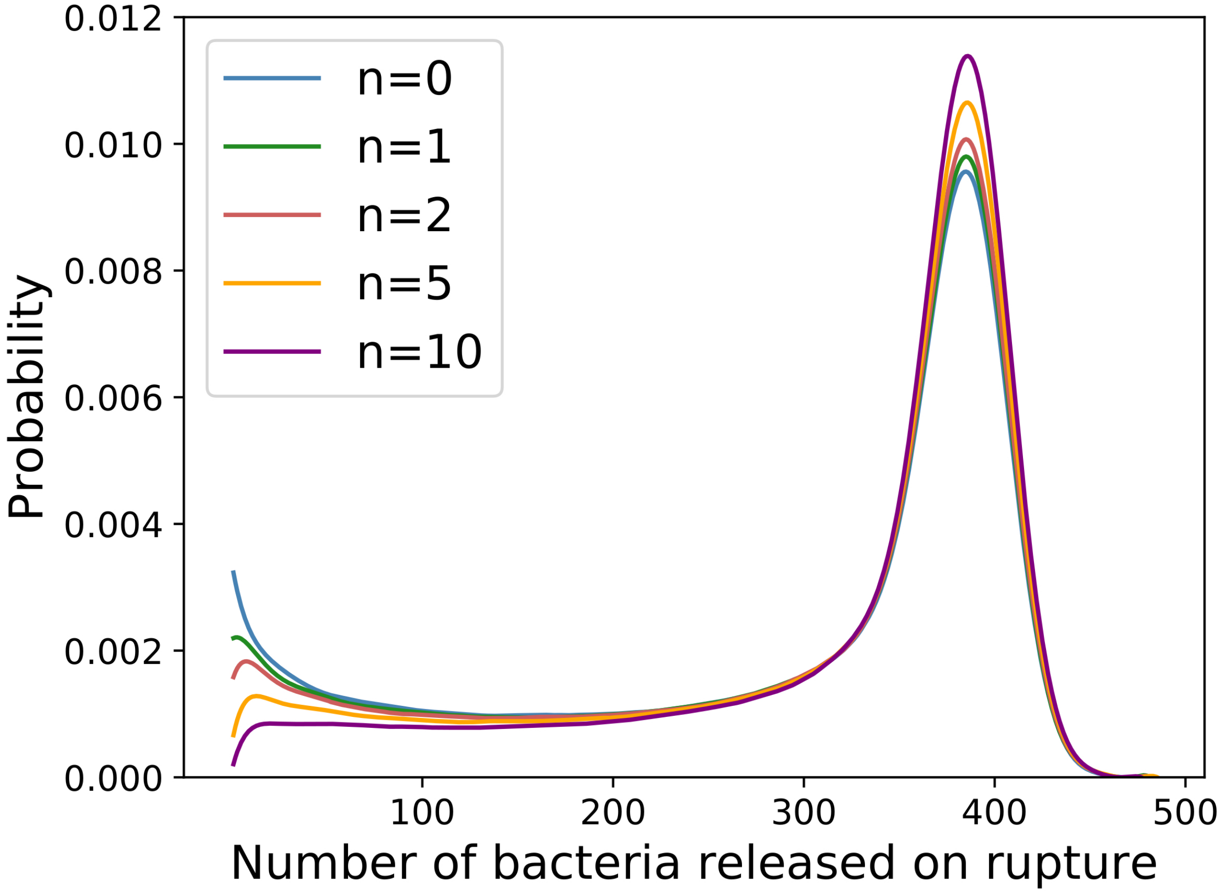

Figure S3. The distribution of the predicted number of bacteria released by a single phagocyte on rupture, when n additional bacterial intake events are allowed to occur in $[0, Median[T^{rupture}]]$. $n = 0$ corresponds to the distribution in Figure 9, where the only bacterium entering into the phagocyte is the one starting the within-phagocyte infection.

Results in Figure S3 suggest that, if a large number of bacteria are allowed to enter into the phagocyte, the rupture size distribution becomes uni-modal, where almost all the infected phagocytes have some intracellular bacterial replication before rupture. This is in contradiction with recent experimental results by Brock and Parmely (2017), where a significant amount of phagocytes are observed to rupture releasing very few bacteria, so that our model seems to support the use of $n = 0$ or very small values of n, representing that each phagocyte is infected by a single bacterium or a very small number of bacteria.

2 ANALYSIS OF THE WITHIN-HOST MODEL

The objective in the within-host model is to compute dose response probabilities $\pi_{(i,j)} = \lim_{t\to\infty} \mathbb{P}\left(\mathbf{Y}(t) = M \,|\, \mathbf{Y}(0) = (i,\, j)\right)$, and restricted mean times $r_{(i,j)} = \mathrm{E}\left[T_{(i,j)} 1_{T_{(i,j)}<+\infty}\right]$ in Carruthers et al. (2018). Dose response probabilities can be obtained by solving the system of equations resulting from Eq. (1) in Carruthers et al. (2018). The space of states of the within-host model (see

(Carruthers et al., 2018, Figure 3))

$$\mathcal{S}_{\mathcal{Y}} \;=\; \{0\} \cup \{(i,j) :\; 1 \leq i \leq M-1,\; 0 \leq j \leq i\} \cup \{M\}$$

can be organised by levels as $\mathcal{S}_{\mathcal{Y}} = \{0\} \cup \bigcup_{j=0}^{M-1} L(j) \cup \{M\}$ where

$$\begin{aligned} L(0) &= \{(i,0) :\; 1 \leq i \leq M-1\}, \\ L(j) &= \{(i,j) :\; j \leq i \leq M-1\}, \quad 1 \leq j \leq M-1. \end{aligned}$$

Thus, by storing probabilities $\pi_{(i,j)}$ in column vectors $\boldsymbol{\pi}_0 = \left(\pi_{(1,0)}, \pi_{(2,0)}, ..., \pi_{(M-1,0)}\right)^T$, and $\boldsymbol{\pi}_j = \left(\pi_{(j,j)}, \pi_{(j+1,j)}, ..., \pi_{(M-1,j)}\right)^T$, $1 \leq j \leq M-1$, Eq. (1) in Carruthers et al. (2018) can be rewritten in matrix form as

$$\begin{pmatrix} \boldsymbol{\pi}_0 \\ \boldsymbol{\pi}_1 \\ \vdots \\ \boldsymbol{\pi}_{M-2} \\ \boldsymbol{\pi}_{M-1} \end{pmatrix} = \begin{pmatrix} \mathbf{G}_{00} & \mathbf{G}_{01} & & & \\ \mathbf{G}_{10} & \mathbf{G}_{11} & \mathbf{G}_{12} & & \\ & \ddots & \ddots & \ddots & \\ & & \mathbf{G}_{M-2,M-1} & \mathbf{G}_{M-2,M-2} & \mathbf{G}_{M-2,M-1} \\ & & & \mathbf{G}_{M-1,M-2} & \mathbf{G}_{M-1,M-1} \end{pmatrix} \begin{pmatrix} \boldsymbol{\pi}_0 \\ \boldsymbol{\pi}_1 \\ \vdots \\ \boldsymbol{\pi}_{M-2} \\ \boldsymbol{\pi}_{M-1} \end{pmatrix} + \begin{pmatrix} \mathbf{c}_0 \\ \mathbf{c}_1 \\ \vdots \\ \mathbf{c}_{M-2} \\ \mathbf{c}_{M-1} \end{pmatrix}.$$

Non-null entries of matrices $\mathbf{G}_{j,j-1}$ are given by:

$$\begin{aligned} (\mathbf{G}_{1,0})_{i,i+k-1} &= \frac{\delta R_k}{(\mu+\alpha)(i-1)+\delta}, \quad i = 1, ..., M-1\,,\; k = 1, ..., M-i, \\ (\mathbf{G}_{j,j-1})_{i-j+1,i-j+1+k} &= \frac{\delta j R_k}{(\mu+\alpha)(i-j)+\delta j}, \quad i = j, ..., M-1,\; j = 2, ..., M-1\,,\; k = 1, ..., M-i. \end{aligned}$$

The non-null entries of $\mathbf{G}_{j,j}$ satisfy

$$\begin{aligned} (\mathbf{G}_{0,0})_{i,i-1} &= \frac{\mu i}{(\mu+\alpha)i}, \quad i = 2, ..., M-1, \\ (\mathbf{G}_{j,j})_{i-j+1,i-j} &= \frac{\mu(i-j)}{(\mu+\alpha)(i-j)+\delta j}, \quad i = j+1, ..., M-1,\; j = 1, ..., M-2, \end{aligned}$$

and if $j = M-1$ we note that the level only consists of a single state and hence $\mathbf{G}_{M-1,M-1} = 0$. Non-null entries of matrices $\mathbf{G}_{j,j+1}$ are given by

$$\begin{aligned} (\mathbf{G}_{\mathbf{0},\mathbf{1}})_{i,i} &= \frac{\alpha i}{(\mu+\alpha)i}, \quad i = 1, ..., M-1, \\ \left(\mathbf{G}_{\mathbf{j},\mathbf{j+1}}\right)_{i-j+1,i-j} &= \frac{\alpha(i-j)}{(\mu+\alpha)(i-j)+\delta j}, \quad i = j+1, ..., M-1,\; j = 1, ..., M-2. \end{aligned}$$

Finally, for vectors $\mathbf{c}_j$, we have that $\mathbf{c}_0 = \mathbf{0}$ and non-null entries for $1 \leq j \leq M-1$ are

$$(\mathbf{c}_j)_{i-j+1} = \frac{\delta j}{(\mu+\alpha)(i-j)+\delta j} \left(\sum_{k \geq M-i+1} R_k \right), \quad i = j, ..., M-1.$$

It is clear that an adapted version of Algorithm 1, not reported here, can be used to solve this matrix equation. Finally, we note that the computation of the restricted mean times $r_{(i,j)} = \mathrm{E}\left[T_{(i,j)} 1_{T_{(i,j)}<+\infty}\right]$, not reported here for the sake of brevity, can be carried out by following similar first-step arguments, and by making use of a similar matrix-oriented approach.

REFERENCES

Brock, S. R. and Parmely, M. J. (2017). Complement c3 as a prompt for human macrophage death during infection with francisella tularensis strain schu s4. *Infection and Immunity* 85, e00424–17

Carruthers, J., López-García, M., Gillard, J. J., Laws, T. R., Lythe, G., and Molina-París, C. (2018). A novel stochastic multi-scale model of *Francisella tularensis* infection to predict risk of infection in a laboratory. *Frontiers Microbiology* 9:1165. doi:10.3389/fmicb.2018.01165

Golovliov, I., Baranov, V., Krocova, Z., Kovarova, H., and Sjöstedt, A. (2003). An attenuated strain of the facultative intracellular bacterium *Francisella tularensis* can escape the phagosome of monocytic cells. *Infection and Immunity* 71, 5940–5950

Gómez-Corral, A. and López-García, M. (2015). Lifetime and reproduction of a marked individual in a two-species competition process. *Applied Mathematics and Computation* 264, 223–245

He, Q.-M. (2014). *Fundamentals of matrix-analytic methods* (Springer)

Osogami, T. and Harchol-Balter, M. (2006). Closed form solutions for mapping general distributions to quasi-minimal PH distributions. *Performance Evaluations* 63, 524–552

Wood, R., Egan, J., and Hall, I. (2014). A dose and time response Markov model for the in-host dynamics of infection with intracellular bacteria following inhalation: with application to *Francisella tularensis*. *Journal of The Royal Society Interface* 11
